# An Extracellular Matrix–Producing Subset of Cancer-Associated Fibroblasts Drives Chemoresistance in Breast Cancer via SRC Activation and G0S2 Upregulation

**DOI:** 10.1158/0008-5472.CAN-25-0966

**Published:** 2025-11-12

**Authors:** Isabella Hofer, Yann Kieffer, Arianna Mencattini, Hugo Croizer, Rana Mhaidly, Stéphanie Descroix, Christophe Le Tourneau, Maud Kamal, Constance Lamy, Claire Bonneau, Paul H. Cottu, Anne Vincent-Salomon, Eugenio Martinelli, Fatima Mechta-Grigoriou, Maria Carla Parrini

**Affiliations:** 1 Institut Curie, Stress and Cancer Laboratory, Equipe labélisée par la Ligue Nationale contre le Cancer, PSL Research University, Paris, France.; 2 Inserm, U1339, CNRS UMR3666, Paris, France.; 3 Department of Electronic Engineering, University of Rome Tor Vergata, Rome, Italy.; 4 Institut Curie, CNRS UMR168, MMBM Team, Institut Pierre-Gilles de Gennes, PSL Research University, Paris, France.; 5 Department of Drug Development and Innovation, Institut Curie, Inserm U1331, Paris-Saclay University, Paris, France.; 6 Department of Oncological Surgery and Inserm U900, 22 Statistical Methods for Precision Medicine, Institut Curie Hospital Group, Université de 23 Versailles Saint Quentin, Yvelines, France.; 7 Department of Medical Oncology, Institut Curie Hospital Group, Université Paris Cité, Paris, France.; 8 Department of Diagnostic and Theranostic Medicine, Institut Curie Hospital Group, Institute of Women’s Cancer, PSL Research University, Paris, France.

## Abstract

Chemotherapy resistance remains a major hurdle for treating patients with triple-negative breast cancer (TNBC). Although cancer-associated fibroblasts (CAF) as an overall population have been shown to modulate treatment response, innovative approaches are required to decipher which and how distinct CAF populations drive chemoresistance. In this study, by combining analysis of data from patients with TNBC with *ex vivo* modeling using tumor-on-chip technology, we identified a specific CAF population, the extracellular matrix–producing myofibroblasts (ECM-myCAF), that mediated resistance to chemotherapy. The proportion of ECM-myCAFs decreased after chemotherapy in chemosensitive patients but remained unchanged in chemoresistant patients. In tumor-on-chip models, primary ECM-myCAFs promoted TNBC cell survival under chemotherapy treatment. Single-cell RNA sequencing, advanced cell imaging, and functional assays showed that ECM-myCAFs activated SRC kinases in TNBC cells, likely through secreted factors, and upregulated the apoptosis regulator G_0_–G_1_ switch 2 (G0S2). SRC inhibition or G0S2 silencing completely abolished TNBC cell chemoresistance driven by ECM-myCAFs. Altogether, this work reveals the unique role of the specific ECM-myCAF population and identifies G0S2 as a key player in chemoresistance in TNBC.

**Significance:**

Integration of patient data with *ex vivo* tumor-on-chip modeling identifies an extracellular matrix–producing myofibroblast population that contributes to chemoresistance and can be targeted to improve outcomes in triple-negative breast cancer.

## Graphical Abstract

**Figure:**
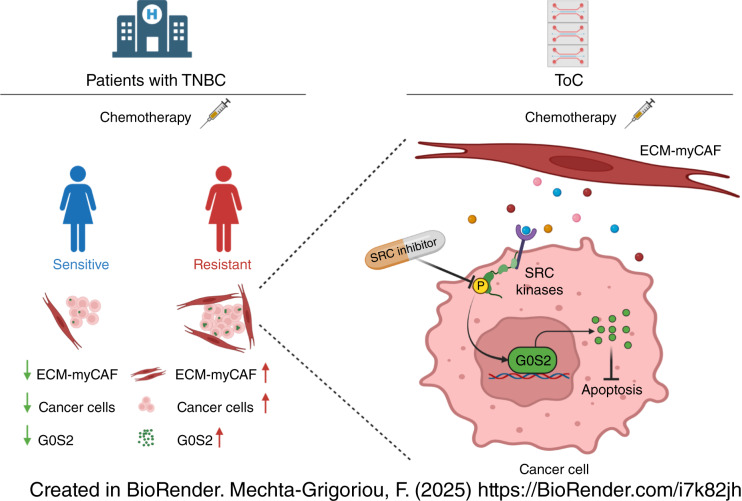

## Introduction

Triple-negative breast cancer (TNBC) is a highly aggressive subtype of breast cancer for which druggable targets remain scarce. Current treatments for patients with TNBC include neoadjuvant chemotherapy (e.g., taxanes such as paclitaxel and anthracyclines such as doxorubicin, cyclophosphamide, and platinum drugs), followed by surgery and radiotherapy. Immunotherapy targeting PD-1/PD-L1 in combination with chemotherapy has been approved as neoadjuvant treatment for high-risk early-stage TNBC; benefits are encouraging but still modest (1). Chemotherapy resistance remains the major challenge in combating TNBC progression and relapse. In this regard, the contribution of the tumor microenvironment (TME) is still poorly understood.

Cancer-associated fibroblasts (CAF) are one of the most abundant TME components. CAFs can be distinguished from normal fibroblasts based on their transcriptional profiles and the expression of specific surface proteins, including fibroblast activation protein (FAP), α-smooth muscle actin (αSMA), or integrin β1 (2). Still, CAFs are a highly heterogeneous population. In many cancer types, in both humans and mice, CAFs have been shown to be composed of several subpopulations that differentially participate in immunosuppression, metastatic spread, and chemoresistance (2–23). Importantly, the two myofibroblastic subsets, FAP^+^ SMA^+^ CAFs (also called CAF-S1) and FAP^−^ SMA^+^ cancer-associated perivascular-like (CAP) fibroblasts (also called CAF-S4), are enriched in aggressive breast cancer subtypes (HER2^+^ and TNBC) and in metastatic lymph nodes (2, 11). In line with these observations, our laboratory found that FAP^+^ SMA^+^ CAFs inhibit antitumor immune responses and promote metastatic spread (2, 11, 24–27). Moreover, by performing one of the most resolutive single-cell RNA sequencing (scRNA-seq) analyses of FAP^+^ SMA^+^ CAFs from breast cancers, we showed that this population is composed of several cellular clusters, including inflammatory (iCAF) and myofibroblastic (myCAF) CAFs, as first detected in pancreatic cancer (3, 7, 17). Based on specific gene expression profiles, these clusters were defined as extracellular matrix-producing (ECM-myCAF), TGFβ signaling pathway, wound healing, IFNαβ-mediated response, actomyosin pathway, detoxification pathway, IL-signaling pathway, and IFNγ-mediated response (10). The ECM-myCAF cluster accumulates in breast cancer enriched in regulatory T-lymphocytes and is associated with primary resistance to immunotherapies in metastatic melanoma and lung cancer (10). Finally, the ECM-myCAF cluster is the most abundant one in tumors before treatment (10) and is spatially localized in the closest proximity to cancer cells in patients with breast cancer (28).

Although the implication of CAFs as a global population in chemotherapy resistance is well established (29), the impact of specific CAF clusters on chemoresistance in patients with TNBC remains poorly understood. Correlations between the enrichment of specific CAF populations and the chemotherapy responses of patients with TNBC have been identified, but the underlying mechanisms have been investigated only in a few cases (30–34). A major limitation to a better mechanistic understanding of the role of CAFs in chemoresistance is the lack of appropriate experimental approaches to model the TME *ex vivo*. Tumor-on-chip (ToC) technology (35–38), which is part of the emerging field of microphysiologic systems, offers an elegant solution to this problem. ToCs reconstitute the human TME in 3D inside microfluidic devices, with precise control of experimental parameters. Previously, we showed that CAF-dependent immunotherapy resistance can be recapitulated in ToCs (39–41). Indeed, ToC is an ideal approach to recapitulate the response to chemotherapies and to study the CAF-dependent chemoresistance mechanisms because (i) the cells are in a 3D environment that better recapitulates *in vivo* drug response than 2D conditions; (ii) the cell composition (identity, density, ratio) can be finely tuned, contrary to spheroid cultures; and (iii) the complex tumor ecosystem dynamics can be visualized in real time.

In this work, we integrated data from samples of patients with TNBC and an *ex vivo* ToC experimental approach with the aim of mechanistically investigating the role of CAFs in TNBC chemotherapy resistance. First, we observed that the ECM-myCAF content decreases after chemotherapy, only in chemosensitive but not in chemoresistant TNBC tumors, suggesting that the ECM-myCAF population may play a key role in chemoresistance through interaction with TNBC cells. To test this hypothesis, we performed functional assays using 3D ToC devices by coculturing primary ECM-myCAFs isolated from breast cancer with TNBC cell lines and treated these ToCs with standard chemotherapies (doxorubicin and paclitaxel). In this way, we highlighted a robust and quantifiable ECM-myCAF–mediated chemoresistance in TNBC cells. By combining bulk RNA-seq data from patients with TNBC before and after treatment, advanced automated imaging, scRNA-seq from ToC-derived cells, and gene silencing in functional assays, we identified the G_0_–G_1_ switch 2 (G0S2) protein, which is upregulated in TNBC cells by ECM-myCAFs. G0S2, a small basic protein of 103 amino acids, was first identified in blood mononuclear cells, putatively playing a role during the cell-cycle transition from the G_0_-phase to the G_1_-phase (42). Since then, G0S2 has been suggested to participate in several cellular processes, including lipid metabolism (43–45), apoptosis (46, 47), cell survival (48), oxidative phosphorylation (49), epithelium-to-mesenchymal transition (EMT), and invasion (48, 50). However, the multifaceted cellular functions of G0S2 are still largely unknown, in particular its role in chemoresistance in TNBC, which we decipher here. Overall, our findings demonstrate the role of ECM-myCAFs in the chemotherapy resistance of TNBC and identify G0S2 as a new key player downstream of SRC family kinase activation.

## Materials and Methods

### Experimental design

In this study, we analyzed transcriptomic data from patients with chemosensitive and chemoresistant TNBC to determine which CAF populations are relevant for resistance to chemotherapy *in vivo*. Using a ToC approach, we recapitulated the *ex vivo* ability of primary ECM-myCAFs to promote the chemoresistance of TNBC cells, and we dissected the underlying molecular mechanisms using advanced live imaging, scRNA-seq, and functional assays.

### SCANDARE biobanking study information

SCANDARE (NCT03017573) is a prospective biobanking study in which samples, including tumor tissues, were collected after written informed consent was obtained from all patients. The study was approved by a national ethics committee (CPP Ile-de-France 3) and the French National Agency for the Safety of Medicines and Health Products in June 2016. All analyses of tumor samples were performed in accordance with national regulations and recognized ethical guidelines, including the Declaration of Helsinki, on the protection of individuals participating in biomedical research. By participating in SCANDARE, patients receive standard treatments and agree to have additional sampling done along their disease evolution. Patient inclusion started in April 2017 with a 5-year follow-up. All patients received standard treatment according to the stage of the disease and usual procedures. Eighty-eight female patients with TNBC, and 114 samples were analyzed. From 52 chemosensitive patients, 62 samples (49 pre- and 13 post-chemotherapy) were analyzed. From 36 chemoresistant patients, 52 samples (29 pre- and 23 post-chemotherapy) were analyzed. The allocation of patients into chemosensitive or chemoresistant groups was assessed by the response to treatment at surgery using the residual cancer burden (RCB) index. Patients with an RCB index of 0 or 1 were categorized as chemosensitive, whereas those with an RCB index of 2 to 3 were defined as chemoresistant.

### Deconvolution of bulk RNA-seq data from the TNBC SCANDARE cohort

Cell-type composition of 114 bulk RNA-seq samples from the retrospective SCANDARE cohort was estimated using BayesPrism version 2.0. A scRNA-seq atlas derived from Croizer and colleagues (28) was used as a reference. Only triple-negative patients were considered for cancer cells. The raw count matrix of 63,374 cells from this cellular atlas was finally used as input for prior information. Labels were derived from the annotations of the 39 cell types and states (28). Mitochondrial and ribosomal protein-coding genes were removed, as well as *MALAT1* and genes from the X and Y chromosomes, following indications from BayesPrism’s authors. Deconvolution was performed only on protein-coding genes to reduce batch effects and speed up computation. Default parameters to control Gibbs sampling and optimization were used. The final estimation of cell-type fraction and inferred gene expression matrix for cancer cells were obtained for the 114 samples and used for downstream analysis. A heatmap showing the global cell-type composition of the 114 bulk RNA-seq samples from the TNBC SCANDARE cohort was created using the pheatmap (RRID: SCR_016418) R package. Hierarchical clustering was applied to both samples and cell types using Euclidean distance and the ward.D2 method. Values were centered and scaled by cell types. Differences in overall cell-type composition according to treatment status and response were assessed using the Pearson *χ*^2^ test. Differences within one specific cell type were evaluated using the two-sided Wilcoxon rank-sum test. Expression of G0S2 and serum amyloid A1 (SAA1) in cancer cells was inferred using the BayesPrism algorithm with the get.exp() function.

### Culture of human TNBC cell lines

Human MDA-MB-231 (RRID: CVCL_0062) and MDA-MB-436 (RRID: CVCL_0623) TNBC cell lines were cultured in DMEM high glucose (Gibco, #41966-029) supplemented with 10% FBS (Biosera, #FB-1001-500), 100 U/mL penicillin, and 100 μg/mL streptomycin (Sigma-Aldrich, #P4333). Cells were passaged once 80% confluency was reached. All cells were maintained in a humidified 20% O_2_ and 5% CO_2_ incubator. Cell identity was verified monthly by using the short tandem repeat DNA profiling (Promega, #B9510) method. Additionally, all cell lines and primary CAFs used in this study were tested for *Mycoplasma* contamination on a monthly basis (qPCR-based; Eurofins, #50400400) and were confirmed to be *Mycoplasma*-free as of June 16, 2025. Where indicated, TNBC cells were treated with either 100 nmol/L doxorubicin (Teva Pharmaceuticals) or different concentrations of dasatinib (Merck/Sigma-Aldrich, #SML2589-50MG), an inhibitor of SRC, LYN, and LCK. Dasatinib was reconstituted in DMSO to a stock concentration of 2 mg/mL (4.098 mmol/L). TNBC cells were seeded in six-well plates (Falcon Pharma, #353046) and allowed to attach for 6 hours; then, media were refreshed with media containing either DMSO (VWR, # A3672.0100) or dasatinib.

### Isolation, culture, and characterization by flow cytometry of primary ECM-myCAF and CAP fibroblasts

Fresh human breast cancer tumors were collected immediately after surgery. All samples were naïve of treatment when we isolated CAFs for *ex vivo* culture. Tumors were cut into approximately 1 mm^3^ tissue pieces and placed onto plastic dishes (Corning, #353003). CAFs were cultivated in DMEM high glucose (Sigma-Aldrich, #D6429), supplemented with 10% decomplemented FBS (Biosera, #FB-1001/500), 100 U/mL penicillin, and 100 μg/mL streptomycin (Sigma-Aldrich, #P4333). Cells were cultivated at 37°C with 1.5% O_2_ and 5% CO_2_. Media were changed every 2 to 3 days. Cells were passaged once 80% confluency was reached and were used for experiments until a maximum passage of 10.

CAF identity was determined by flow cytometry after amplification after isolation and prior to ToC generation. Cells were washed with 1× PBS (Gibco, #D8537) prior to detachment with TrypLE (Fisher Scientific, #11568856) for 5 minutes at 37°C with 20% O_2_ and 5% CO_2_. Cells were collected in DMEM high glucose (Sigma-Aldrich, #D6429), supplemented with 10% decomplemented FBS (Biosera, #FB-1001/500), 100 U/mL penicillin, and 100 μg/mL streptomycin (Sigma-Aldrich, #P4333), followed by centrifugation at 1,200 rpm for 5 minutes. Cells were stained with a cocktail of anti–FAP-APC (1:100, R&D Systems, #MAB3715), anti–SDC1-BUV737 (1:25, BD Biosciences, #612834), and anti–LAMP5-PE (1:10, Miltenyi Biotec, #130-109-156) for 25 minutes at room temperature in the dark. Then, PBS+ (= 1× PBS, supplemented with 10% FBS and 2 mmol/L EDTA, Invitrogen, #15575020) was added to each sample prior to centrifugation at 1,500 rpm for 5 minutes. Resulting cell pellets were resuspended in PBS+ and analyzed using the BD LSRFortessa Cell Analyzer. CAP fibroblasts have been isolated from human breast cancers and cultivated in TPP tissue culture plates (TPP, #92406) and pericyte medium (CliniSciences, cat. #1201-SC) to maintain their identity, as previously described (2, 11).

### ToC preparation

Microfluidic chips were purchased from AIM-Biotech (#289DAX-1). Cells were prepared as previously described by Veith and colleagues (40). In brief, TNBC cells (MDA-MB-231 or MDA-MB-436) were stained with 5 μmol/L CellTrace Yellow (Invitrogen, #C34567) according to the manufacturer’s protocol. Then, TNBC cells ± primary ECM-myCAFs were seeded in the central chamber of DAX-1 chips embedded in a matrix composed of type I rat tail collagen (Gibco, #A1048301) at a final concentration of 2.3 mg/mL. ToC generation was performed in a cold room at 4°C to prevent premature gel polymerization. The density of cancer cells is 4,200 cells/μL. The density of CAFs is 1,200 cells/μL. The ratio of 3.5 cancer cells to one CAF was used to mimic the *in vivo* situation, as observed in samples from patients with TNBC. After loading cells into the designated channel, ToC devices were incubated for 30 minutes at 37°C with 20% O_2_ and 5% CO_2_ in a humidified chamber to allow collagen polymerization. Afterward, 125 μL of DMEM high glucose (Sigma-Aldrich, #D6429), supplemented with 10% FBS (Biosera, #FB-1001/500), 100 U/mL penicillin, and 100 μg/mL streptomycin (Sigma-Aldrich, #P4333), was added to each lateral chamber. ToCs were incubated for 6 to 15 hours at 37°C with 20% O_2_ and 5% CO_2_ prior to replacing the media, which contained 3 μmol/L CellEvent Caspase-3/7 Detection Reagent (Invitrogen, #C10423) ± 2 μmol/L doxorubicin (Teva Pharmaceuticals) or 1 μmol/L paclitaxel (Fresenius Kabi) and/or ±500 nmol/L Dasatinib. Then, ToCs were transferred into the incubating chamber 37°C with 20% O_2_ and 5% CO_2_ of the live imaging microscope.

### Live cell imaging

Live cell imaging was performed as previously described (40, 41). Briefly, time-lapse images were acquired with an inverted Leica DMi8 equipped with a Retiga R6 camera and Lumencor SOLA SE 365 light engine using a 5× objective. The filter cubes used were TXRed (excitation filter 560/40 nm, emission filter 630/75 nm, dichroic mirror 585 nm) and GFP (excitation filter 470/40 nm, emission filter 525/50 nm, dichroic mirror 495 nm). The video microscope was equipped with a motorized stage for multipositioning acquisition and a CO_2_ and temperature-controlled (37°C) incubator. Distilled water was added to the plastic wells between gel inlets of the DAX-1 chips. Additionally, sterilized and humidified small sponges were placed within the incubator chamber of the microscope to maintain a humidified atmosphere. As in the AIM-Biotech devices the gas permeability is provided by the underside sealing layer; before inserting them on the microscope stage, we placed them on standard microscope glass slides and lifted them using magnet holders (1 mm thick) to create an air circulation space underneath the devices for CO_2_ control. The acquisition of images in transmission and fluorescent channels was performed every 30 minutes for a total duration of up to 72 hours. Three positions per gel/condition were acquired.

### Image analysis

Manual video analysis was performed using ImageJ software (RRID: SCR_003070).

Survival (in %) was calculated every 10-hour time window as follows:

$$\left(\frac{\text{Number}\;\text{of}\;\text{alive}\;\text{cells}\;\text{at}\;t_{0}\;-\;\text{Number}\;\text{of}\;\text{apoptotic}\;\text{cells}\;\text{between}\;t_{0}\;\text{and}\;t_{n}}{\text{Number}\;\text{of}\;\text{alive}\;\text{cells}\;\text{at}\;t_{0}}\right)\;\times \;100$$

where t_0_ is the time of acquisition start and t_n_ is the time window start.

The apoptosis rate (in %) was calculated every 10-hour time window as follows:

$$\left(\frac{\text{Number}\;\text{of}\;\text{apoptotic}\;\text{cells}\;\text{between}\;t_{n}\;\text{and}\;t_{n+10h}}{\text{Number}\;\text{of}\;\text{alive}\;\text{cells}\;\text{at}\;t_{n}}\right)\;\times \;100$$

### Automatic analysis of cancer death

We used the Spatio Temporal Apoptosis MaPper (STAMP) method (40, 41), with some modifications described here. For cell localization, we applied a shallow detection approach as tumor cells are highly static within gels (red channel). The method is based on an adaptive Otsu segmentation method, also called the Bradley method (51). The method applies binarization to every pixel in the image by computing the threshold as the local mean intensity around the neighborhood of the pixel (here, the neighborhood size is equal to 1/8th of the image size). A pixel is then claimed as belonging to a tumor cell if it is assigned to a tumor cell in at least 95% of the time frames along the video. Then, a binary image, or “tumor mask,” is assigned to the video, labeling tumor cells in white and the background in black. To separate the cell region (foreground) and the surrounding region (cell background), we applied morphologic operators to each segmented tumor region (52). By dilating each cell region by a factor of r_dilate_ and then subtracting the original region from the dilated region, we were able to separate the foreground region from the cell background region. The quantity r_dilate_ is fixed here as one third of the square root of the area of each cell. In this way, the larger the cell region, the larger the background area. In order to extract the green emission signals of each tumor cell (i.e., tumor apoptosis events), we transposed the positions of tumor cells from the red to the green channel video. Compared with the original STAMP, assuming negligible cell movement, the extraction of each green signal is simplified as it is not necessary to estimate the green signal from a moving region but rather from a fixed region over time.

### Tumor CAF proximity analysis

Tumor cells were detected and tracked by applying the STAMP method (40, 41). CAFs were detected in the transmission channel (acquired with a higher time resolution, 5 minutes/frame) by applying a semantic segmentation (53) approach based on a convolutional neural network. Such an approach trains a network using grayscale or color images paired with the corresponding ground truth images (i.e., black and white images in which the object to be detected is manually segmented). In this work, we utilized a pretrained InceptionResNetV2 architecture, which underwent a fine-tuning step using a set of 126 image crops. Each crop contained a CAF that was manually segmented by biologists. We defined and computed the following six parameters:

i) Number of contacts between tumor and CAF cells. For each tumor cell, this parameter is the total count of times a CAF is located at a distance of less than 18 μm from the *C*_i_ centroid of the tumor cell.
ii) Minimum tumor-CAF distance. For tumor cells without direct contacts with CAFs (distance greater than 18 μm), this parameter is the minimum distance between the closest CAF boundary and the tumor cell *C*_i_ centroid, computed over all CAFs at each time point (every 5 minutes). Therefore, we have one value for each tumor cell at each time point.
iii) Intersection over union (IOU) at 82 μm. IOU is a measure of closeness between objects having a generally nonpunctual shape. Indicating with *A* and *B* the two shapes, it is calculated as the intersection of *A* and *B* (*A* ∩ *B*), namely the area in common, divided by the union of the two shapes (*A* ∪ *B*).

$$IOU\;=\;\frac{A\;\cap B}{A\cup B}$$

We applied the IOU concept to quantify the tumor surrounding area occupied by CAFs at a given time. In detail, indicating with $A_{CAF}$ the area covered by the CAF and with $A_{R}$ the circular areas having a radius equal to 82 μm, we calculated one value for each tumor cell and for each time as follows:

$$IOU1\;=\;\;\frac{A_{CAF}\;\cap A_{R}}{A_{CAF}\cup A_{R}},$$

### scRNA-seq analysis on ToC

After 42 hours of TNBC and ECM-myCAF mono- or coculture, media were removed from lateral channels. After two washes with 1× PBS to remove residual serum, 20 μL of collagenase I (Millipore, #SCR103), at a concentration of 4 mg/mL in DMEM/F-12 (Gibco, #11320033), supplemented with 25 μg/mL of DNAse (Roche, #11284932001), was added to each lateral channel. ToCs were placed back into the incubator for 30 minutes to facilitate gel digestion. To collect cells, each lateral channel was flushed with 100 μL of DMEM high glucose (Gibco, #41966-029) supplemented with 10% FBS (Biosera, #FB-1003-500) and passed through a 50-μm cell strainer (Sysmex Corporation, #04-004-2327) to remove residual undigested collagen. To achieve a sufficient yield of viable cells, six gels per condition (MDA-MB-231 monoculture, ECM-myCAF monoculture, or MDA-MB-231/ECM-myCAF coculture) were pooled into a single tube after digestion. Cell suspensions were centrifuged at 1,500 rpm for 10 minutes. Dead cells and debris were removed using the MACS Dead Cell Removal Kit (Miltenyi Biotec, #130-090-101) according to the manufacturer’s protocol. Cells were centrifuged at 1,500 rpm for 10 minutes and diluted to a final concentration of 1,000 cells/μL in nuclease-free water (Invitrogen, #AM9937). Single-cell capture, lysis, and cDNA library construction were performed using the following: Chromium Next GEM from 10x Genomics, using the Chromium Next GEM Single Cell 3′ GEM, Library & Gel Bead Kit version 3.1 (10x Genomics, #1000121), Chromium Next GEM Chip G Single Cell Kit (10x Genomics, #1000120), and Chromium Next GEM Single Cell 3′ Library Construction Kit version 3.1 (10x Genomics, #1000157). The generation of gel beads-in-emulsion, barcoding, post–gel beads-in-emulsion reverse transcription cleanup, and cDNA amplification were performed according to the manufacturer’s protocol. cDNA quality and quantity were assessed on the Agilent 2100 Bioanalyzer using the Agilent High Sensitivity DNA Kit (Agilent, #5067-4626), and library construction was followed according to 10x Genomics instructions. Sequencing was facilitated using next-generation sequencing Illumina short read and NovaSeq with a sequencing depth of 50,000 reads per cell.

Processing of raw data, including demultiplexing of raw base call files into FASTQ files, alignment, filtering, barcode, and unique molecular identifier counting, was performed using the 10x Cell Ranger pipeline version 6.0.0. Reads were aligned to the *Homo sapiens* (human) genome assembly GRCh38 (hg38). Seurat version 4.4.0 was used for log normalization, scaling, dimensionality reduction, and clustering using default parameters. Cells with less than 1,000 features detected and a mitochondrial fraction higher than 25% were excluded. Integration of sequencing runs was performed using Harmony version 1.0.3 to mitigate batch effects. Data were visualized using Uniform Manifold Approximation and Projection (UMAP). Thirty dimensions were used. Label transfer using the FindTransferAnchors function from Seurat was used to annotate CAFs and cancer cells. The scRNA-seq atlas from Croizer and colleagues (28) was used as a reference. Differentially expressed genes between mono- and coculture were identified for CAFs and cancer cells separately using the FindAllMarkers function from Seurat with the following parameters: logfc.threshold = 0.1, test.use = “wilcox,” and min.pct = 0.01. Genes with an adjusted *P* value < 0.05 were considered differentially expressed and used for functional enrichment analysis. Functional enrichment for upregulated genes upon coculture or monoculture in CAF and MDA-MB-231 cells was performed using the Metascape (RRID: SCR_016620) web-based portal (54). Inference of transcription factor (TF) activity from the gene expression of their target (regulon) was assessed by using the VIPER version 1.32 and DoRothEA version 1.10 R packages. Only regulons with a high confidence level (A, B, and C) were included in the analysis.

### Transwell cultures

TNBC cells were seeded in a 12-well plate (Falcon Pharma, #353043). Per well, 50,000 MDA-MB-231 or 100,000 MDA-MB-436 cells were seeded in DMEM high glucose (Gibco, #41966-029), supplemented with 10% FBS (Biosera, #FB-1001-500). TNBC cells were incubated at 37°C with 20% O_2_ and 5% CO_2_ for 6 hours to first allow cell adhesion. Then, ECM-myCAFs were seeded in DMEM high glucose (Gibco, #41966-029) at a 1:1 ratio to TNBC cells in the upper chamber of a 12-well cell culture insert (Falcon Pharma, #353182). Cells were cocultured and/or treated with 100 nmol/L doxorubicin for either 0, 20, 40, 42, or 70 hours at 37°C with 20% O_2_ and 5% CO_2_ prior to protein extraction.

### Protein extraction and Western blot

Cells were washed with ice-cold 1× PBS (Gibco, #D8537) and lysed by adding ice-cold lysis buffer containing 50 mmol/L Tris HCl (Invitrogen, #15567-027), 150 mmol/L NaCl (Invitrogen, #AM9760G), 1% IGEPAL (Sigma-Aldrich, #CA-630), 0.1% SDS (Invitrogen, #AM9822), 1 mmol/L EDTA (Invitrogen, #15575-038), 50 mmol/L NaF (Sigma-Aldrich, #450243-10G), and 1 mmol/L sodium orthovanadate (Sigma-Aldrich, #S7920-100G), freshly supplemented with 1× Halt Phosphatase Inhibitor Cocktail (Thermo Fisher Scientific, #784236), 1× Halt Protease Inhibitor Cocktail (Thermo Fisher Scientific, #87785), and 0.05% sodium deoxycholate (Sigma-Aldrich, #30970-25G). Cells were scraped and incubated on ice for 20 minutes. Lysates were collected, sonicated in 30-second cycles for 10 minutes, and centrifuged at 13,000 rpm at 4°C for 10 minutes. Protein concentration was determined by Bradford assay (Bio-Rad, #5000006). Lysis buffer and 4x Laemmli Sample Buffer (Bio-Rad, #1610747) were added to equalize the protein amount for each sample.

Proteins (10–20 μg) were loaded into NuPAGE 4% to 12% Bis-Tris gels (Invitrogen, #NP0321BOX and #WG1402BOX). Protein migration was performed for 1.5 hours at 150 V in 1× NuPAGE MES SDS Running Buffer (Invitrogen, #NP002). Proteins were then transferred to a 0.45-μm nitrocellulose membrane (GE HealthCare, #10600002) and incubated overnight at 4°C with the appropriate primary antibodies: 1:50,000 β-actin (Proteintech, t#66009-1-Ig, RRID: AB_2687938), 1:1,000 G0S2 (Proteintech, cat. #12091-1-AP, RRID: AB_2877824), and 1:2,000 SRC (Cell Signaling Technology, #2110, RRID:AB_10691385) in Tris-buffered saline-Tween 20 (TBST; TBS tablets: EUROMEDEX, #EU1-7500-100; Tween 20: Sigma-Aldrich, #P2287) supplemented with 5% nonfat dry milk (Cell Signaling Technology, #999S) or 1:2,000 phospho-SRC Tyr^416^ (Cell Signaling Technology, cat. #6943, RRID: AB_10013641) in TBST supplemented with 5% BSA (EUROMEDEX, # 04-100-812-C). On the next day, membranes were washed three times with TBST and incubated with the appropriate peroxidase-conjugated secondary antibodies (Jackson ImmunoResearch Laboratories, #115-035-003). Proteins were visualized using enhanced chemiluminescence detection (Thermo Fisher Scientific, SuperSignal West Pico PLUS Chemiluminescent Substrate, #34580 for visualization of actin, SRC, and pSRC Tyr^416^; SuperSignal West Atto Ultimate Sensitivity Chemiluminescent Substrate, Thermo Fisher Scientific, #A38555 for G0S2). To reprobe the same membranes with different primary antibodies, membranes were stripped for 10 minutes with antibody stripping buffer (CliniSciences, # ST010) and subsequently washed with distilled water for 30 minutes. Then, membranes were blocked again for 1 hour in TBST containing either 5% milk powder or 5% BSA prior to reprobing. Analyses of immunoblots were performed using ImageJ software (RRID: SCR_003070).

### Silencing by siRNA

For siRNA-mediated silencing of G0S2, 1 × 10^6^ MDA-MB-231 or 2 × 10^6^ MDA-MB-436 cells were seeded in 100 mm tissue culture dishes (Falcon Pharma, #353003) and subsequently transfected with a pool of four different siRNAs, each at 5 nmol/L (ON-TARGET plus Human G0S2 siPool, target sequences: CCA-AGG-AGA-UGA-UGG-CCC​A, GCA-CUA-GGG-AGG-AAG-GAU​A, GAG-AAA-CCG-CUG-ACA-UCU-A, GGG-AAG-AUG-GUG-AAG-CUG​U, Dharmacon, #SO-3183100G). As controls, TNBC cells were seeded at the same densities and transfected with ON-TARGET plus nontargeting siRNA at a final concentration of 20 nmol/L (target sequence: UGG-UUU-ACA-UGU-UGU-GUG-A, Dharmacon, #D-001810-02-05). Transfections were performed with DharmaFECT 2 Transfection Reagent (Horizon Discovery, #T-2005-01) according to the manufacturer’s instructions. Silencing efficacy was analyzed 48, 72, 96, and 120 hours after transfection by Western blot. For ToC experiments, transfected cells were incubated for 44 hours at 37°C with 20% O_2_ and 5% CO_2_ in DMEM supplemented with 10% FBS (Biosera, #FB-1001/500), prior to detachment and subsequent ToC generation.

### Multiplex kinase activity assay

After 42 hours of mono- or cocultures, transwell inserts were removed, and 12-well plates with TNBC cells were kept on ice, washed twice with ice-cold 1× PBS (Gibco, #D8537), and lysed using M-PER Mammalian Extraction Buffer (Thermo Fisher Scientific, #78503), supplemented with 1× Halt Phosphatase Inhibitor Cocktail (Thermo Fisher Scientific, #784236) and 1× Halt Protease Inhibitor Cocktail (Thermo Fisher Scientific, #87785). Cells were scraped and incubated on ice for 15 minutes. Protein lysates were collected and centrifuged at 13,000 rpm for 15 minutes at 4°C. Supernatants were transferred to a fresh tube, snap-frozen in liquid nitrogen, and sent for analysis to PamGene International BV (https://pamgene.com). In short, protein lysates were loaded on protein tyrosine kinase and serine/threonine kinase PamChip arrays that were spotted with peptides embedded within a porous 3D membrane, allowing for high-throughput profiling of cellular kinase activity. The data workflow consisting of image quantification, quality control, statistical analysis, visualization, and interpretation was performed using the BioNavigator software (https://pamgene.com/ps12/).

### Statistics

All statistical analyses and graphical representations of data were performed in the R environment (version 4.2.0, https://cran.r-project.org, RRID: SCR_003005) or using GraphPad Prism (RRID: SCR_002798) software (version 9.4.1). Statistical tests used are in agreement with data distribution: Normality was first checked using the Shapiro–Wilk test, and parametric or nonparametric two-sided tests were applied according to normality, as indicated in each figure legend.

## Results

### ECM-myCAF content decreases after chemotherapy in chemosensitive but not in chemoresistant patients with TNBC

**Figure 1.**
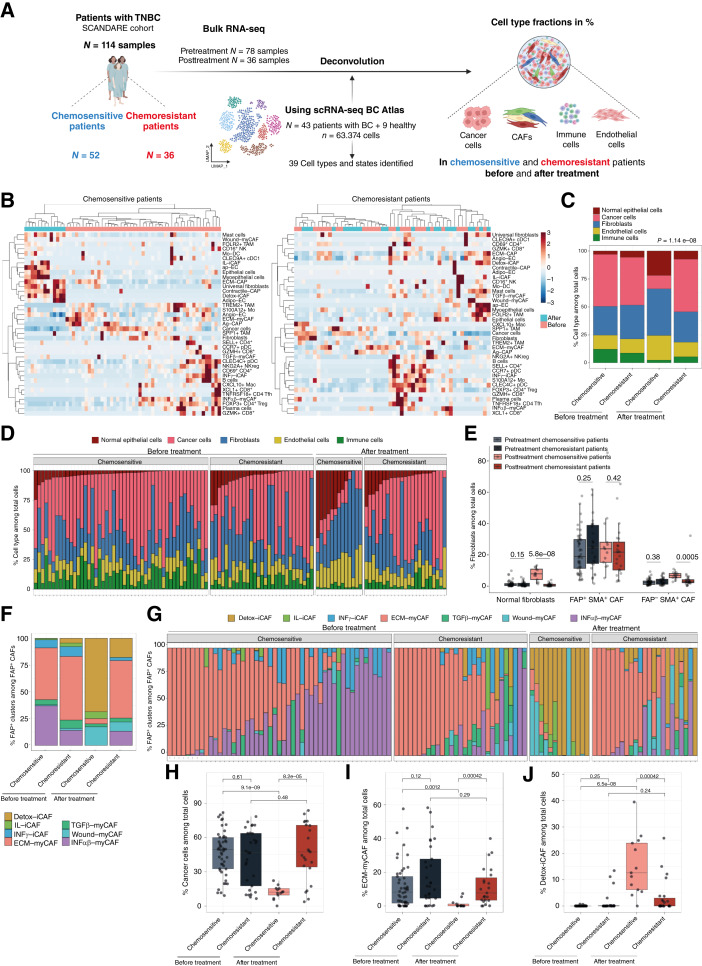
Cell-type distribution before and after chemotherapy in chemosensitive and chemoresistant patients with TNBC. **A,** Flowchart illustrating the patient cohort, sample acquisition, data processing, and analysis. BC, breast cancer. **B,** Heatmap showing the cell-type proportions in pre- and post-chemotherapy tumor samples (*N* = 114 samples) from the TNBC SCANDARE Curie cohort, as inferred by deconvolution of bulk RNA-seq data using a scRNA-seq–based atlas derived from Croizer and colleagues (28). Left, *N* = 62 samples from 52 chemosensitive patients. Right, *N* = 52 samples from 36 chemoresistant patients. The response to treatment was assessed at surgery by the RCB index. Patients with an RCB index of 0 or 1 were categorized as chemosensitive, whereas those with an RCB index of II to III were defined as chemoresistant. Hierarchical clustering was applied to both samples and cell types using Euclidean distance and the ward.D2 method. Values were centered and scaled by cell types. **C,** Distribution of normal epithelial, cancer, fibroblastic, endothelial, and immune cells among all cell types according to chemotherapy response (*N* = 114 samples). **D,** Percentages of normal epithelial, cancer, fibroblastic, endothelial, and immune cells among total cells according to chemotherapy response (*N* = 114). **E,** Distribution of normal fibroblasts, FAP^+^ SMA^+^ CAF, and FAP^−^ SMA^+^ CAF among total cells in chemosensitive and chemoresistant patients before and after treatment (*N* = 114). **F,** Distribution of FAP^+^ CAF clusters among all FAP^+^ CAF according to chemotherapy response (*N* = 114). **G,** Percentages of the different FAP^+^ CAF clusters among FAP^+^ CAF in chemosensitive and chemoresistant patients before and after chemotherapy (*N* = 114). Each column represents an individual patient. **H–J,** Percentages of cancer cells (**H**), ECM-myCAF (**I**), and detoxification iCAF (**J**) among total cells in chemosensitive and chemoresistant patients before and after chemotherapy (*N* = 114). **C,***P* value from the Pearson *χ*^2^ test; **E **and **H–J,***P *values from two-sided Wilcoxon rank-sum test. **A,** Created in BioRender. Mechta-Grigoriou, F. (2025) https://BioRender.com/oxvjiu9.

We first compared the cellular composition of the TME between chemosensitive and chemoresistant patients with TNBC (Fig. 1A). To do so, we deconvoluted bulk RNA-seq and estimated the proportions of the different cell types that composed TNBC. We analyzed a retrospective cohort of 88 patients with TNBC (52 chemosensitive and 36 chemoresistant) composed of 114 samples (78 pre- and 36 post-chemotherapy; see Supplementary Table S1 for a detailed description of the TNBC SCANDARE Curie cohort). The cellular composition of each sample was inferred by deconvolution of bulk RNA-seq data by applying BayesPrism (55) and using a breast cancer cellular atlas based on a highly resolutive scRNA-seq dataset (28) as a reference. This atlas was composed of 63,374 cells isolated from patients with breast cancer, which enabled us to distinguish 39 different cell types and states (Supplementary Fig. S1A and S1B). Unsupervised clustering estimated the fractions of different cell types and states, as inferred by deconvolution of bulk RNA-seq data (Fig. 1B). Interestingly, this analysis highlighted how treatment response correlates with changes in cellular composition and revealed clear disparities between chemosensitive and chemoresistant patients with TNBC (Fig. 1B; Supplementary Fig. S1C). Indeed, pre- and posttreatment samples were clearly segregated in chemosensitive patients but not in chemoresistant patients, suggesting that chemotherapy substantially modified TME cellular composition in chemosensitive patients (Fig. 1B, left) but had almost no impact in chemoresistant patients (Fig. 1B, right). Considering the proportion of epithelial (normal and cancer), fibroblastic, immune, and endothelial cells in each sample, we observed a strong increase in the CAF fraction in chemosensitive patients after treatment, concomitant with a decrease in the fraction of cancer cells (Fig. 1C and D). We also observed an increase in endothelial and normal epithelial cells, as expected in chemosensitive patients; still, the impact on fibroblasts was the most pronounced (Fig. 1C). We thus next investigated the impact of chemotherapy on CAF heterogeneity in these patients. The increased fibroblastic fraction in chemosensitive TNBC tumors after chemotherapy was associated with a global enrichment in normal-like fibroblasts and FAP^−^ SMA^+^ cancer–associated perivascular-like fibroblasts, whereas the overall proportion of FAP^+^ SMA^+^ CAFs remained high before and after treatment, regardless of patient response (Fig. 1E). As the proportion of FAP^+^ CAFs reached around 25% of total cells on average and was much higher than other fibroblast populations (Fig. 1E), we sought to further characterize these FAP^+^ CAFs at the resolution of clusters (10). Interestingly, we observed significant differences in the content of FAP^+^ CAF clusters following treatment, indicating the need to study variations in FAP^+^ CAFs at the cluster level (Fig. 1F–J). Before treatment, TNBC predominantly accumulated myCAF clusters, in particular ECM-myCAFs, in both chemosensitive and chemoresistant patients (Fig. 1F and G). After chemotherapy, a striking difference was observed in the composition of FAP^+^ CAF clusters. In chemosensitive patients (showing a significant decrease in cancer cell content, as expected, Fig. 1H), the proportion of ECM-myCAFs decreased dramatically (Fig. 1F, G, and I) in favor of detoxification iCAFs (Fig. 1F, G, and J). In contrast, the ECM-myCAF content remained stable and high in chemoresistant patients (Fig. 1F, G, and I). This was also confirmed by paired comparison at the individual patient level (Supplementary Fig. S1D). Altogether, these observations show that the ECM-myCAF content significantly decreases in chemosensitive but not in chemoresistant patients, suggesting ECM-myCAFs may play a key role in resistance to treatment in patients with TNBC.

### Primary ECM-myCAFs protect TNBC cells from doxorubicin and paclitaxel treatments in a 3D ToC model

**Figure 2.**
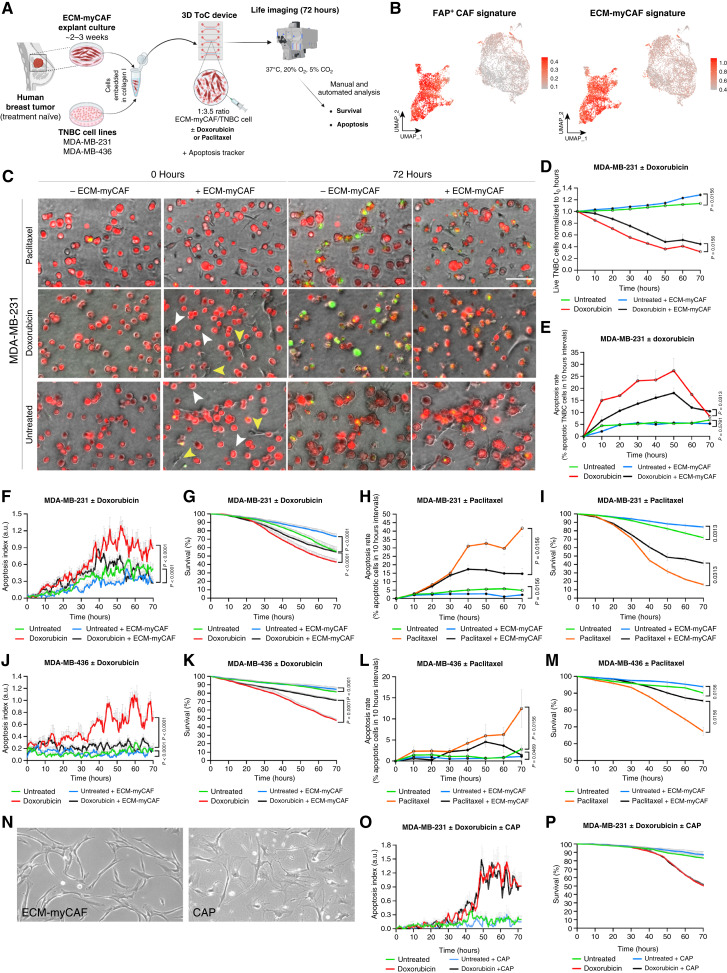
Primary ECM-myCAFs protect TNBC cell lines from death under doxorubicin and paclitaxel treatment in a 3D ToC model. **A,** Schematic overview of 3D ToC generation from primary breast cancer–derived ECM-myCAF and TNBC cell lines. **B,** UMAP showing high expression of FAP^+^ CAF (also referred to as CAF-S1) and ECM-myCAF gene signatures (10) in ECM-myCAF, validating their identity after 3D ToC culture assessed by scRNA-seq (*n* = 9,651 cells from two independent experiments using two different patient-derived ECM-myCAFs). **C,** Representative images of MDA-MB-231 ± ECM-myCAF ± doxorubicin or paclitaxel treatment at acquisition start (0 hours) and 72 hours after culture in ToC. Scale bar, 100 μm. White arrows, cancer cells; yellow arrows, ECM-myCAFs. **D,** Manual quantification of live MDA-MB-231 ± ECM-myCAF ± doxorubicin normalized to live cells at acquisition start (0 hours; *n* = 3 independent experiments using three different patient-derived ECM-myCAFs; *n* = 3 videos per condition). **E,** Manual quantification of MDA-MB-231 apoptosis ± ECM-myCAF ± doxorubicin (*n* = 3 independent experiments using three different patient-derived ECM-myCAFs; *n* = 3 videos per condition). **F** and **G,** Automated quantification of MDA-MB-231 apoptosis index (**F**) and survival (**G**) ± ECM-myCAF ± doxorubicin (*n* = 4 independent experiments using four different patient-derived ECM-myCAFs; *n* = 3 videos per condition). **H** and **I,** Manual quantification of MDA-MB-231 apoptosis index (**H**) and survival (**I**) ± ECM-myCAF ± paclitaxel (*n* = 3 independent experiments using three different patient-derived ECM-myCAFs; *n* = 3 videos per condition). **J** and **K,** Automated quantification of MDA-MB-436 apoptosis index (**J**) and survival (**K**) ± ECM-myCAF ± doxorubicin (*n* = 2 independent experiments using two different patient-derived ECM-myCAFs; *n* = 3 videos per condition). **L** and **M,** Manual quantification of MDA-MB-436 apoptotic index (**L**) and survival (**M**) ± ECM-myCAF ± paclitaxel (*n* = 2 independent experiments using two different patient-derived ECM-myCAFs; *n* = 3 videos per condition). **N,** Representative images displaying typical ECM-myCAF and CAP fibroblast morphology. **O** and **P,** Automated quantification of MDA-MB-231 apoptosis index (**O**) and survival (**P**) ± CAP ± doxorubicin (*n* = 3 videos per condition). All data are represented as the mean ± SEM. Statistical differences were assessed by the Wilcoxon matched-pair signed-rank test. **A,** Created in BioRender. Mechta-Grigoriou, F. (2025) https://BioRender.com/fcjaht2.

We took advantage of an *ex vivo* ToC approach (39, 41, 56) to test the impact of ECM-myCAFs on TNBC cell chemoresistance. To do so, we cocultured primary ECM-myCAFs isolated from patients with breast cancer together with TNBC cell lines (MDA-MB-231 and MDA-MB-436) in a 3D biomimetic collagen matrix inside ToC devices (Fig. 2A). We used our established protocols (10, 28, 57) to specifically isolate and amplify ECM-myCAFs and validated their identity by flow cytometry. As expected, after isolation and amplification, primary ECM-myCAFs were FAP^+^ integrin β1^+^ SDC1^+^ LAMP5^−^ (Supplementary Fig. S2A). We extracted cells from the ToC culture and performed scRNA-seq analysis. After quality control, 9,651 cells were analyzed. Dimensionality reduction followed by UMAP revealed a clear separation of KRT18^+^ MDA-MB-231 TNBC cells from FAP^+^ ECM-myCAFs (Supplementary Fig. S2B). After ToC culture, ECM-myCAFs remained highly positive for FAP^+^ CAF (also referred to as CAF-S1) and ECM-myCAF gene signatures (Fig. 2B). In ToC cocultures, the cancer cell-to-CAF ratio was set at 3.5:1, which very closely mimicked the *in vivo* situation. Indeed, we precisely calculated the cancer cells to ECM-myCAFs ratio, taking advantage of the SCANDARE cohort of 114 patients with TNBC using the deconvoluted RNA-seq data (Supplementary Fig. S1C). The average cancer cells to ECM-myCAFs ratio was 3.4:1 (3.4 cancer cells for 1 ECM-myCAF) for all patients, with a ratio of 4:1 in chemosensitive patients and 3:1 in chemoresistant patients. A red live fluorescent dye (CellTrace) was used to selectively prestain cancer cells and distinguish them from unstained ECM-myCAFs. A green live fluorescent dye (CellEvent Caspase-3/7) was used to monitor apoptosis (Fig. 2C; Supplementary Fig. S2C). Alive and dying cells were counted both manually and automatically using the STAMP algorithm (40). Cancer cells proliferate slowly in 3D; the doubling time of MDA-MB-231 cells was estimated to be around 15 days (Fig. 2D), whereas in 2D dishes, it was ∼24 hours. Of note, in patients, the breast tumor doubling times were estimated by ultrasonography and mammography (58). Although highly heterogeneous, the range was from 10 to 100 days (58), with a mean *in vivo* doubling time estimated at 103 ± 43 days for TNBC (59). Therefore, the slow proliferation in ToCs better mimicked *in vivo* situations than fast-growing cells in 2D dishes. We next evaluated the impact of standard chemotherapies administered to patients with TNBC, including both doxorubicin (for Adriamycin) and paclitaxel (for taxanes). As expected, the addition of doxorubicin to ToC medium efficiently killed MDA-MB-231 cancer cells (Supplementary Videos S1–S4). Strikingly, the addition of ECM-myCAFs significantly increased the number of alive TNBC cells (Fig. 2D) and significantly decreased their apoptosis rate (Fig. 2E). Therefore, this *ex vivo* ToC model demonstrated a chemoprotective effect of ECM-myCAFs on TNBC cells upon doxorubicin treatment. The STAMP algorithm (40) allowed for a more exhaustive, unbiased, temporally resolved, and statistically robust analysis of videos from independent experiments using different patient-derived ECM-myCAFs. Automated STAMP analyses confirmed that the presence of ECM-myCAFs reduced TNBC cell apoptosis (Fig. 2F) and increased TNBC cell survival (Fig. 2G), both in the absence and presence of doxorubicin. Similar experiments were performed with paclitaxel (Supplementary Videos S5 and S6) and showed that ECM-myCAFs additionally protected TNBC cells from paclitaxel-induced apoptosis (Fig. 2H and I). All these findings have been validated with a second TNBC cell line, MDA-MB-436, treated with doxorubicin (Fig. 2J and K) and paclitaxel (Fig. 2L and M), respectively (Supplementary Videos S7–S12). Finally, the specificity of the chemoprotective effect of ECM-myCAFs was demonstrated by performing ToC coculture experiments with another primary CAF population, the FAP^−^ SMA^+^ CAP fibroblasts (also called CAF-S4 in previous studies; Fig. 2N; refs. 2, 11, 57). These CAP fibroblasts did not have any effect on TNBC cell apoptosis (Fig. 2O) and survival (Fig. 2P), both in the absence and presence of doxorubicin. In conclusion, using several primary ECM-myCAFs isolated from independent patients with breast cancer, two TNBC cell lines (MDA-MB-231 and MDA-MB-436), and two standard chemotherapy drugs (doxorubicin and paclitaxel), we highlighted a robust, specific, and highly reproducible chemoprotective effect exerted by the ECM-myCAF population on TNBC cells in *ex vivo* ToC devices.

### “The kiss-of-life”: chemoprotection by ECM-myCAFs occurs at early time points and at short distances from cancer cells

**Figure 3.**
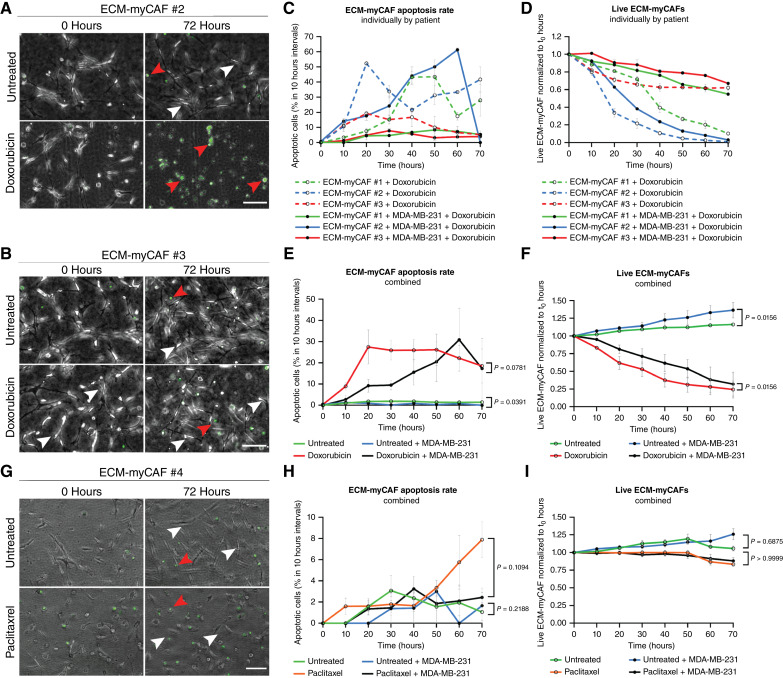
Primary ECM-myCAFs display a heterogeneous response to doxorubicin. **A** and **B,** Representative images of ECM-myCAFs from patient #2 (**A**) and patient #3 (**B**) ± doxorubicin at acquisition start (0 hours) and 72 hours after culture in ToC. Scale bar, 100 μm. White arrows, living cells; red arrows, dead cells. **C,** ECM-myCAF (from three different patients) apoptosis rate ± MDA-MB-231 ± doxorubicin (*n* = 3 independent experiments; *n* = 3 videos per condition). **D,** Alive ECM-myCAF (from three different patients) ± MDA-MB-231 ± doxorubicin normalized to alive cells at acquisition start (0 hours; *n* = 3 independent experiments; *n* = 3 videos per condition). **E** and **F,** ECM-myCAF apoptosis rate and alive cells (combining the three patients) ± MDA-MB-231 ± doxorubicin. **G,** Representative images of ECM-myCAFs under paclitaxel treatment at acquisition start (0 hours) and 72 hours after culture in ToC. Scale bar, 100 μm. White arrows, living cells; red arrows, dead cells. **H,** Manual quantification of ECM-myCAF apoptosis rate (combining three patients) ± MDA-MB-231 ± paclitaxel. **I,** Alive ECM-myCAF (combining three patients) ± MDA-MB-231 ± paclitaxel normalized to alive cells at acquisition start (0 hours). Data are represented as the mean ± SEM. Statistical differences were assessed by the Wilcoxon matched-pair signed-rank test.

We also analyzed the effects of chemotherapy on ECM-myCAFs (Supplementary Videos S13–S16). Some ECM-myCAFs were very sensitive to doxorubicin and completely dead after 72 hours (e.g., patient #2, Fig. 3A), whereas others were very resistant (e.g., patient #3, Fig. 3B). The quantifications of apoptotic (Fig. 3C) and alive (Fig. 3D) ECM-myCAFs under doxorubicin treatment showed very different drug sensitivity from one patient to another. The reasons for this drug response heterogeneity remained unclear, as our previous studies had shown that ECM-myCAF transcriptional identity is conserved in culture among breast cancer subtypes and across multiple donors when using the same method of isolation (10, 28). In addition, there was no correlation between the patient donor clinical outcomes and the chemosensitivity of their ECM-myCAFs in culture. Notably, despite this heterogeneity, the presence of MDA-MB-231 cells decreased ECM-myCAF death (Fig. 3E) and significantly increased the number of alive ECM-myCAFs (Fig. 3F), both in the absence and presence of doxorubicin, indicating mutual protection between ECM-myCAFs and TNBC cells. In contrast, paclitaxel strongly affected ECM-myCAF motility and morphology (Fig. 3G) but had a very mild impact on their apoptosis and survival rate (Fig. 3H and I).

**Figure 4.**
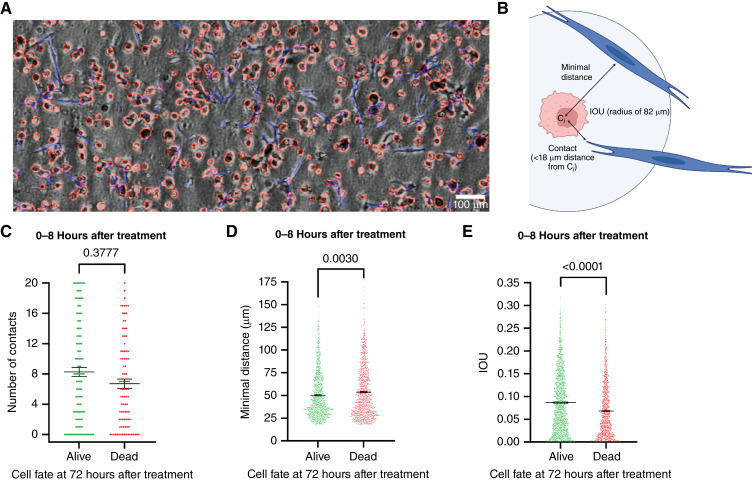
“Kiss of life” from ECM-myCAFs protects TNBC cells from doxorubicin. **A,** Representative image of MDA-MB-231 (red) and ECM-myCAF (blue) automatically detected. Scale bar, 100 μm. **B,** Measurements assessed by the algorithm: number of contacts (ECM-myCAF at minimal distance <18 μm from the centroid of the TNBC cell, C_i_); minimal distance between individual TNBC cells and ECM-myCAFs; and area covered by ECM-myCAFs in a circle with a radius of 82 μm around the TNBC cell (IOU). **C,** Number of contacts at early time points (from treatment start to 8 hours after treatment), with respect to cancer cell fate (dead or alive; *n* = 92–135 measurements). **D,** Minimal distance at early time points, with respect to cancer cell fate (dead or alive; *n* = 1,109–1,420 measurements). **E,** IOU in the time interval at early time points, with respect to cancer cell fate (dead or alive at 72 hours after treatment; *n* = 795–1,341 measurements). Data are represented as individual values, including mean ± SEM. Statistical differences were assessed by the Kolmogorov–Smirnov test. **B,** Created in BioRender. Mechta-Grigoriou, F. (2025) https://BioRender.com/i42a245.

Despite interpatient heterogeneity among primary fibroblast donors, all tested ECM-myCAFs showed a similar magnitude of chemoprotection on TNBC cells in ToCs, independent of their own responses to chemotherapies. This suggests that the chemoprotective effect of ECM-myCAFs is mediated very early during the coculture with cancer cells. To test this priming hypothesis, we developed an image analysis method for ToC videos, based on the use of a convolutional neural network, to automatically measure the number of physical contacts and the distances between each TNBC and ECM-myCAF cell, as well as the area occupied by ECM-myCAF in proximity to each individual cancer cell (IOU; Fig. 4A and B; Supplementary Video S17). Measurements were taken at early time points after doxorubicin treatment (0–8 hours) and correlated with the fate of cancer cells (dead or alive) at 72 hours after treatment. There was no difference in the number of direct physical contacts between cancer cells and ECM-myCAFs at early time points about the cancer cell state (alive or dead) at 72 hours after treatment (Fig. 4C). In contrast, TNBC cells that remained alive after treatment showed shorter minimum distances to ECM-myCAFs at treatment start compared with cancer cells that ultimately died (Fig. 4D). Moreover, the area around TNBC cells covered by ECM-myCAFs at early time points was higher for cancer cells that remained alive at late time points compared with those that finally died (Fig. 4E). These results indicate that the proximity of ECM-myCAFs at early time points is a key priming event promoting the chemoresistance of TNBC cells. We termed this event “the kiss-of-life,” meaning that ECM-myCAFs prime, at early time points, TNBC cells to become chemoresistant.

### Combining transcriptomic data from ToC and patients with TNBC identifies G0S2 as a potential player in ECM-myCAF–mediated chemoresistance of cancer cells

**Figure 5.**
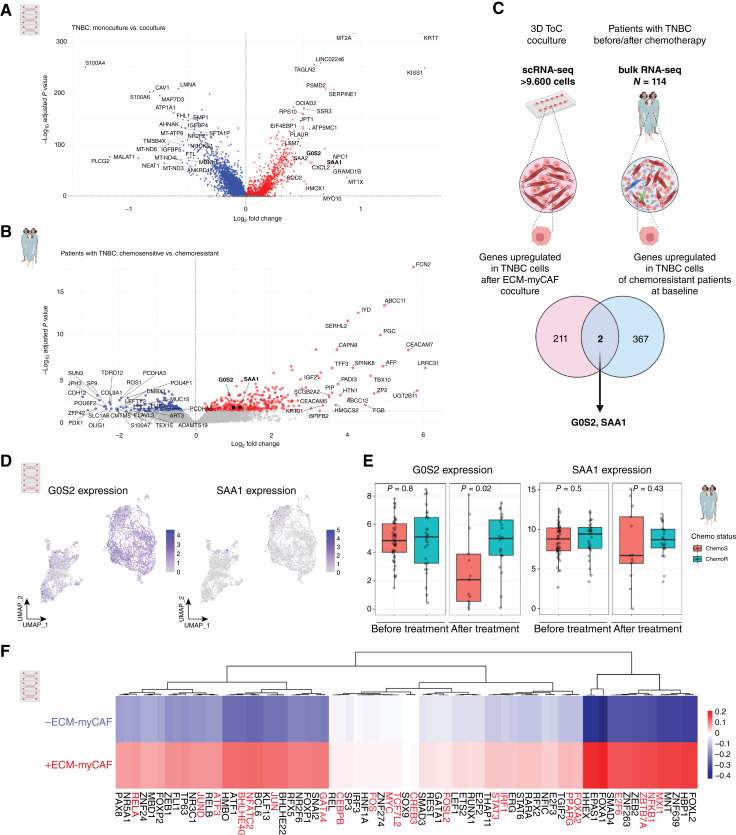
Combining transcriptomic datasets from ToC and patients with TNBC reveals G0S2 as a candidate involved in ECM-myCAF–mediated chemoresistance. **A,** Volcano plot of differentially expressed genes in MDA-MB-231 after ToC coculture with ECM-myCAFs (compared with monoculture). Genes significantly upregulated in MDA-MB-231 upon ECM-myCAF coculture are shown in red, genes significantly downregulated are shown in blue, and genes with nonsignificant expression changes are shown in gray. **B,** Volcano plot of differentially expressed genes in patients with chemosensitive vs. chemoresistant TNBC at baseline. Genes significantly upregulated in chemoresistant patients are highlighted in red. Genes significantly upregulated in chemosensitive patients are highlighted in blue. Genes with nonsignificant expression changes are depicted in gray. **C,** Venn diagram showing the overlap between the genes upregulated in MDA-MB-231 cells after coculture with ECM-myCAFs in ToC and those upregulated in TNBC cells in chemoresistant compared with chemosensitive patients at baseline. **D,** UMAP from scRNA-seq data (*n* = 9,651 total cells; 3,866 ECM-myCAFs and 5,785 MDA-MB-231) showing G0S2 expression (left) and SAA1 expression (right). **E,** G0S2 expression (left) and SAA1 expression (right) in cancer cells of patients with TNBC. **F,** Heatmap of TF activities (analyzed using DoRothEA) in TNBC cells after mono- or coculture with ECM-myCAF in ToC. TFs that are known to regulate G0S2 transcription (according to the Signaling Pathways Project web knowledgebase) are depicted in red. **C,** Created in BioRender. Mechta-Grigoriou, F. (2025) https://BioRender.com/p69mf1k.

We investigated the mechanism by which ECM-myCAFs prime TNBC cells by comparing the transcriptomic scRNA-seq profiles of TNBC cells in the presence or absence of ECM-myCAFs in ToC devices. The analysis of 9,651 cells (Supplementary Fig. S2B) enabled us to study transcriptomic changes induced upon coculture of TNBC cancer cells with ECM-myCAFs in ToCs. Interestingly, 211 genes were upregulated in TNBC cells upon coculture with ECM-myCAFs (Fig. 5A). Functional enrichment analyses showed that these genes were mainly enriched in response to stress, oxidative phosphorylation, and the mitochondrial electron transport chain (Supplementary Fig. S3A–S3D). We next hypothesized that TNBC chemoresistance observed *ex vivo* in ToC, upon coculture with ECM-myCAFs, may recapitulate molecular mechanisms that also occur *in vivo* in patients with chemoresistant TNBC. We therefore compared bulk RNA-seq data from TNBC chemoresistant and chemosensitive patients (SCANDARE Curie cohort; Supplementary Table S1). To do so, we inferred the transcriptomic profiles of TNBC cells from patients by performing deconvolution of bulk RNA-seq at baseline before chemotherapy treatment. We found 367 upregulated genes in cancer cells of patients with chemoresistant TNBC compared with those with chemosensitive TNBC (Fig. 5B). These gene functions are implicated in cholesterol and lipid metabolic processes and the regulation of hormone levels (Supplementary Fig. S3E and S3F). Interestingly, two genes (*G0S2* and *SAA1*) were identified in both the *ex vivo* (ToC) and *in vivo* (patients) transcriptomic analyses. Indeed, these two genes were upregulated in TNBC cells upon coculture with ECM-myCAFs in ToC and at baseline in chemoresistant TNBC compared with chemosensitive patients (Fig. 5C). *G0S2* expression was highly expressed in scRNA-seq data from cancer cells in ToC devices, in contrast to *SAA1*, which was barely detected (Fig. 5D). Moreover, *G0S2* expression was strongly upregulated in chemoresistant patients compared with chemosensitive patients after treatment, whereas *SAA1* was not (Fig. 5E). Thus, we focused our attention on the *G0S2* gene, previously reported to regulate apoptosis (46, 47), cell survival (48), EMT (48, 50), and mitochondrial processes (49).

Finally, we took advantage of the scRNA-seq datasets from ToC-derived cells to infer TF activity from the expression of TF-target genes using the DoRothEA algorithm (60). We identified 70 TFs, in which activity was increased in TNBC cells upon coculture with ECM-myCAFs (Fig. 5F). Interestingly, among them, 21 TFs are known to regulate *G0S2* transcription according to the Signaling Pathways Project web knowledgebase (61). These findings suggested that *G0S2* transcription was directly upregulated in TNBC cells in the presence of ECM-myCAFs and that G0S2 might be a key player in ECM-myCAF–mediated chemoresistance of TNBC cells.

### G0S2 upregulation in cancer cells is required for ECM-myCAF–mediated chemoprotection

**Figure 6.**
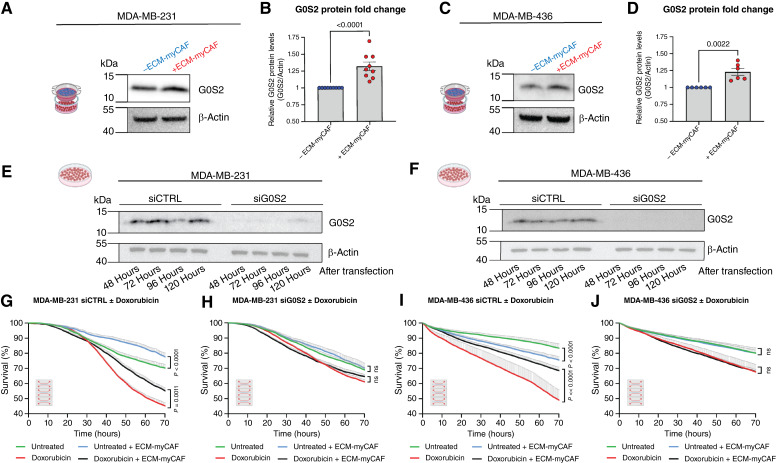
G0S2 knockdown in TNBC cells abolishes the ECM-myCAF–mediated chemoprotective effect. **A,** Representative Western blots showing G0S2 (11 kDa) and β-actin (42 kDa) levels in MDA-MB-231 cells in the absence of or after 42 hours of ECM-myCAF transwell coculture. **B,** Quantification of **A**. G0S2 protein levels in TNBC cells in coculture with ECM-myCAFs are normalized to G0S2 levels in monoculture and are presented as the mean ± SEM. Several Western blots from *n* = 3 independent experiments. **C** and **D,** As in **A** and **B** but for MDA-MB-436 cells. **E,** Western blot showing G0S2 protein levels in MDA-MB-231 cells silenced (siG0S2) or not (siCTRL) for G0S2 at 48, 72, 96, and 120 hours. **F,** As in **E** but for MDA-MB-436 cells. **G,** Automated quantification of MDA-MB-231 siCTRL survival ± ECM-myCAFs ± doxorubicin (*n* = 2 independent experiments using two different patient-derived ECM-myCAFs; *n* = 3 videos analyzed per condition). **H,** Automated quantification of MDA-MB-231 siG0S2 survival ± ECM-myCAFs ± doxorubicin (*n* = 2 independent experiments using two different patient-derived ECM-myCAFs; *n* = 3 videos analyzed per condition). **I** and **J,** As in **G** and **H** but using MDA-MB-436 cells (*n* = 3 independent experiments using three different patient-derived ECM-myCAFs; *n* = 3 videos analyzed per condition). All data are represented as the mean ± SEM. **B,***P *value from the Mann–Whitney test; for video analyses, statistical differences were assessed by the Wilcoxon matched-pair signed-rank test.

We next checked whether G0S2 triggers TNBC chemoresistance. We first assessed whether the coculture of TNBC cells with ECM-myCAFs increased G0S2 at the protein level in addition to the mRNA level. ToCs are miniaturized devices containing only a few thousand cells, a number that is not compatible with Western blot analysis. We thus performed transwell cocultures, with ECM-myCAFs seeded in the upper chamber and TNBC cells in the lower chamber, enabling specific protein analyses of TNBC cells cultured in the presence of ECM-myCAFs. The total G0S2 protein amount was significantly increased in TNBC cells upon coculture with ECM-myCAF with respect to monoculture in both MDA-MB-231 (Fig. 6A and B) and MDA-MB-436 (Fig. 6C and D) cells, suggesting that ECM-myCAF secreted factors mediate G0S2 protein upregulation in cancer cells without physical contact. In addition, we also performed coculture in standard 2D dishes and then sorted the two cell populations by FACS before Western blot analysis, further confirming the upregulation of G0S2 protein in TNBC cells upon coculture with ECM-myCAFs (Supplementary Fig. S4). These results indicate that the “kiss of life” is effective across a wide range of distances, including close cell-to-cell proximity (2D culture), several tens of microns (3D ToC devices), up to about 2 mm (transwells).

We next evaluated the chemoprotective effect of ECM-myCAFs in G0S2-silenced TNBC cells. The silencing was very efficient, starting from 48 hours and stable for at least 120 hours after transfection in TNBC cells (Fig. 6E and F). No obvious effect of *G0S2* silencing was observed on the viability, proliferation, or morphology of TNBC cells. Two days after transfections, TNBC cells were cultured inside ToCs, with or without ECM-myCAFs, in the presence or absence of chemotherapy drugs to test the impact of *G0S2* silencing on the chemoprotective capacities of ECM-myCAFs (Fig. 6G–J). Automated STAMP analysis showed that as observed in nontransfected cells (Fig. 2H), ECM-myCAFs increased the survival of TNBC cells (transfected with nontargeting siRNA, siCTRL) upon treatment, thus protecting TNBC cells (both MDA-MB-231 and MDA-MB-436) from chemotherapy (Fig. 6G and I). Interestingly, when G0S2 was silenced in TNBC cells (siG0S2), the presence of ECM-myCAFs had no effect on the survival of siG0S2 TNBC cells (both MDA-MB-231 and MDA-MB-436) in the absence or presence of chemotherapy (Fig. 6H and J), indicating that G0S2 is required for ECM-myCAF–mediated chemoprotection. Taken as a whole, these results indicate that G0S2 is involved in the TNBC cell response to chemotherapy and is a key player in ECM-myCAF–mediated chemoresistance.

### Role of SRC kinases in *G0S2* upregulation in cancer cells

**Figure 7.**
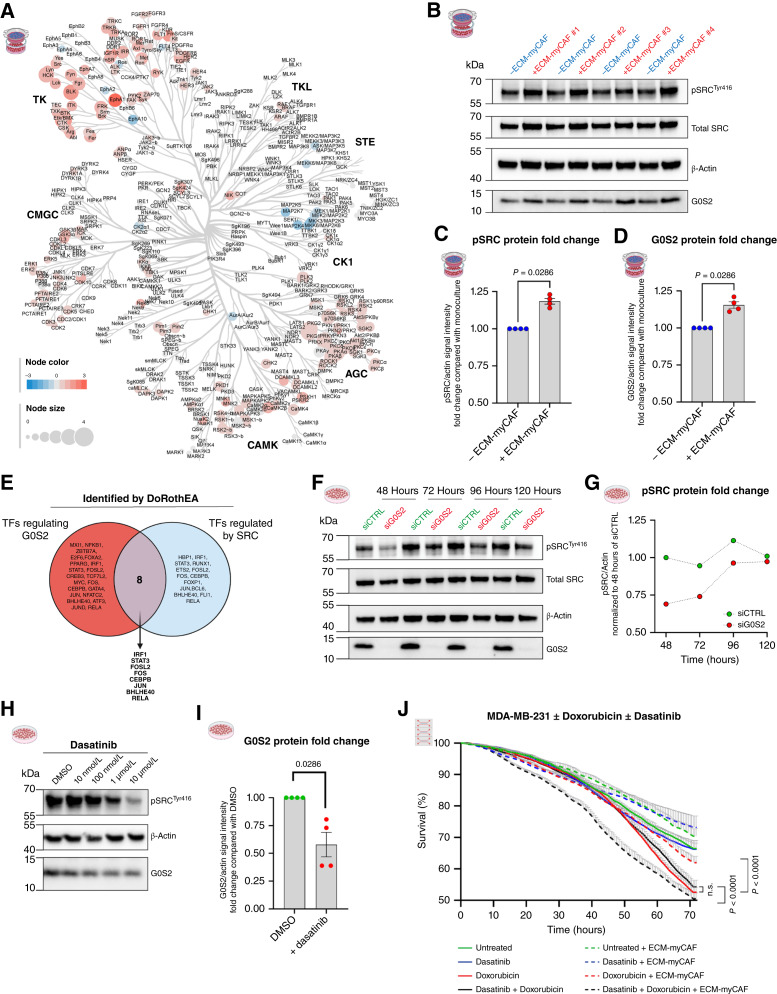
ECM-myCAFs induce SRC kinase activity in TNBC cells. **A,** Kinome tree depicting upregulation and downregulation of kinase family activities in MDA-MB-231 cells upon 42 hours of ECM-myCAF transwell coculture (*n* = 2 different patient-derived ECM-myCAFs). **B,** Western blot showing pSRC^Tyr416^, total SRC, G0S2, and β-actin protein levels in MDA-MB-231 cells after 42 hours of transwell culture without or with ECM-myCAFs (*n* = 4 different patient-derived ECM-myCAFs). **C** and **D,** Quantification of pSRC and G0S2 levels from **B**. pSRC and G0S2 protein levels in MDA-MB-231 in coculture with ECM-myCAF are normalized to their respective protein levels in monoculture. Values are represented as the mean ± SEM. **E,** Venn diagram showing the overlap between TFs predicted to be activated in MDA-MB-231 cells upon ECM-myCAF coculture in ToC and those known to positively regulate *G0S2* transcription (as shown in Fig. 5F), together with TFs identified by Ji and colleagues (62) as regulated by v-SRC. **F,** Western blot showing pSRC, total SRC, G0S2, and β-actin protein levels at indicated time points in MDA-MB-231 cells silenced (siG0S2) or not (siCTRL) for G0S2. **G,** Quantification of **F**. pSRC level was normalized to siCTRL at 48 hours. **H,** Representative Western blot of MDA-MB-231 cells after treatment with increasing concentrations of the SRC family kinases inhibitor dasatinib. **I,** Quantification of G0S2 protein levels upon 48 hours of dasatinib (50 nmol/L) treatment normalized to DMSO control (*n* = 2 independent experiments). Values are represented as the mean ± SEM. **J,** Automated quantification of MDA-MB-231 survival ± ECM-myCAFs ± doxorubicin ± dasatinib (*n* = 3 independent experiments using three different patient-derived ECM-myCAFs; *n* = 3 videos analyzed per condition). **C, D, **and **I,** Statistical significance was assessed by the Mann–Whitney test; **J,***P* values from two-sided Wilcoxon rank-sum test. **E,** Created in BioRender. Mechta-Grigoriou, F. (2025) https://BioRender.com/l3pzirw.

As G0S2 plays a key role in chemoresistance in TNBC cells, we next sought to determine the mechanisms leading to its upregulation in cancer cells in the presence of ECM-myCAFs by performing a pan-kinase screen on TNBC cells. TNBC cells were cultured without or with ECM-myCAFs isolated from two patients with breast cancer. The corresponding TNBC cell lysates were analyzed using the PamGene multiplex kinase assay, which covers the majority of the human kinome (∼350 kinases). Several phosphotyrosine kinases and serine/threonine kinases were found to be differentially regulated in MDA-MB-231 or MDA-MB-436 cells upon coculture with ECM-myCAFs with respect to monoculture (Supplementary Fig. S5A–S5H). Interestingly, the integration of hits for MDA-MB-231 (Fig. 7A) and for MDA-MB-436 cells (Supplementary Fig. S5I) pointed out a converging, statistically robust upregulation of most members of the SRC family kinases (SRC, YES, LYN, HCK, LCK, BLK, FYN, FGR) in both TNBC cell lines in the presence of ECM-myCAFs. In transwell assays, coculture with ECM-myCAFs significantly increased the phosphorylation of the SRC protein (pSRC^Tyr16^, an indicator of SRC activation) in TNBC cells, in addition to the upregulation of the G0S2 protein (Fig. 7B–D). Time course analysis indicated that both pSRC and G0S2 protein levels increased with time in culture, both stimulated consistently by the presence of ECM-myCAFs. The G0S2 protein level reached a peak at 40 hours (Supplementary Fig. S6A–S6F). As a previous work has experimentally established a list of TFs that are activated by v-SRC (62), we compared this list with the list of TFs we identified as being upregulated in TNBC cells upon coculture with ECM-myCAFs (Fig. 5F). By this way, we identified eight common TFs (IRF1, STAT3, FOSL2, FOS, CEBPB, JUN, BHLHE40, RELA; Fig. 7E) that may be involved in the stimulation of *G0S2* transcription downstream of SRC kinases in cancer cells in the presence of ECM-myCAFs. Interestingly, we also observed a decrease in pSRC upon G0S2 silencing, indicating a potential positive feedback loop: SRC phosphorylation positively regulates G0S2 levels, and G0S2 positively regulates SRC phosphorylation (Fig. 7F and G; Supplementary Fig. S6G and S6H).

Then, we tested the effects of SRC kinase inhibition. We treated MDA-MB-231 cells with dasatinib, a drug known to inhibit the kinase activity of SRC family members, such as SRC itself, LCK, or LYN. Increasing doses of dasatinib (from 10 nmol/L to 10 μmol/L) led to a dose-dependent decrease in pSRC^Tyr16^, confirming SRC inhibition (Fig. 7H). Interestingly, dasatinib treatment (50 nmol/L) significantly decreased G0S2 protein levels (Fig. 7I), supporting the role of SRC kinase activity in G0S2 regulation in TNBC cells. We next investigated the effects of dasatinib and doxorubicin, alone or in combination, on TNBC cell survival in the presence and absence of ECM-myCAFs (Fig. 7J). Consistent with our previous observations, ECM-myCAFs protected TNBC cells against doxorubicin. Dasatinib alone had no impact on TNBC monocultures, and its combination with doxorubicin did not enhance cytotoxicity beyond doxorubicin alone. Most importantly, SRC inhibition by dasatinib completely abolished the protective effect of ECM-myCAFs, restoring chemosensitivity to doxorubicin, thus supporting the role of SRC kinase activity in chemoresistance driven by ECM-myCAFs.

**Figure 8.**
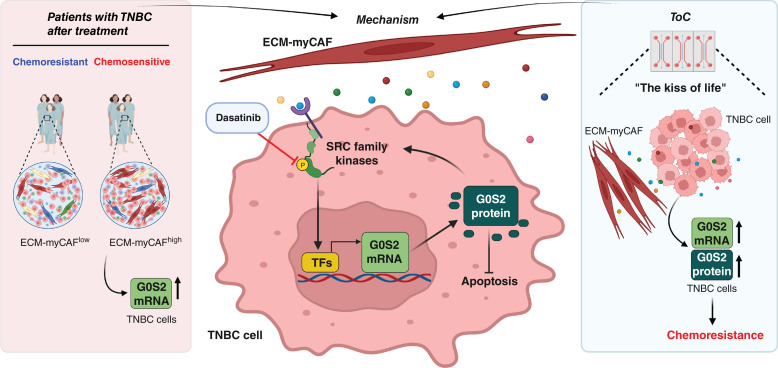
Mechanistic model. A mechanistic model for ECM-myCAF–promoted TNBC chemoresistance emanates from the integration of *in vivo* (in patients) and *ex vivo* (in ToCs) results. Secreted ECM-myCAF factors increase the activity of SRC family kinases in TNBC cells, leading to the upregulation of TFs that positively regulate *G0S2* transcription; the subsequent increase in G0S2 protein reduces cancer cell apoptosis, increases cell survival, and ultimately leads to resistance to chemotherapy treatment. Created in BioRender. Mechta-Grigoriou, F. (2025) https://BioRender.com/v79c085.

In conclusion, all these results led us to propose a G0S2-dependent mechanism by which ECM-myCAFs mediate chemoprotection in TNBC cells (Fig. 8). ECM-myCAFs activate SRC kinases in TNBC cells likely through secreted factors; in turn, SRC family kinases stimulate specific TFs that positively regulate *G0S2* transcription. The subsequent increase in G0S2 protein levels reduces cancer cell apoptosis and ultimately leads to resistance to chemotherapy drugs.

## Discussion

Patients with TNBC are often diagnosed in young women and still have a poor prognosis. Despite the recent introduction of immunotherapy targeting the PD-1/PD-L1 axis, cytotoxic chemotherapies remain the main treatment for patients with TNBC. However, many patients will develop resistance to chemotherapy. At early stages, around 50% of patients with TNBC who have no complete response to neoadjuvant chemotherapy experience a relapse within 3 years, and stage IV TNBC has a median overall survival of less than 2 years (63). Therefore, there is an urgent medical need to elucidate the mechanisms of chemotherapy resistance. Although previous studies have identified intrinsic mechanisms of resistance attributable to cancer cells themselves, very little is known about the impact of the TME, particularly the different CAF populations, on chemotherapy resistance in patients with TNBC. Here, by exploiting an original combination of cutting-edge technologies, we elucidate the role of a specific population of ECM-myCAFs, decipher their cross-talk with TNBC cells, and uncover the underlying mechanism dependent on the G0S2 protein.

Most studies addressing resistance mechanisms in patients with TNBC undergoing neoadjuvant chemotherapy have primarily focused on cancer cell–intrinsic mechanisms. Numerous mechanisms of resistance have been linked to genomic and/or nongenomic properties of TNBC cells, including alterations in multiple signaling pathways, acquisition of cancer stem cell features, and transporter-mediated drug efflux (64, 65). A recent study shows that the expression of chemoresistance genes is driven by TNBC super-enhancers and TFs across TNBC subtypes (66). Still, there is growing evidence that both tumor cell–intrinsic and microenvironmental factors contribute to chemoresistance. In particular, a high stromal content is associated with a poor prognosis, with the most pronounced effect observed in TNBC (66). Moreover, a gene signature of reactive stroma predicts resistance to neoadjuvant chemotherapy in breast cancer (67), with paracrine effects from stroma promoting tumor cell proliferation. In addition, the tumor stroma has also been shown to promote cancer cell proliferation and accelerate cancer cell recovery from stressful conditions caused by chemotherapy cycles, thereby limiting tumor elimination (31). Although the role of CAFs in chemotherapy resistance is now established when considering CAFs as a global population (29), the impact of specific CAF populations is far from being understood.

In our work, we first considered it crucial to identify the CAF population involved in TNBC chemoresistance to achieve clinically relevant results and conclusions. We identified a specific FAP^+^ ECM-producing CAF population (referred to as ECM-myCAFs) as a key player in chemoresistance in patients with TNBC. To do so, we benefited from the SCANDARE Curie cohort of patients with information on long-term clinical responses to chemotherapy treatments. This analysis showed that ECM-myCAF content specifically decreases after chemotherapy in patients with chemosensitive but not in chemoresistant TNBC. Interestingly, a previous study in patients with high-grade serous ovarian cancer using multiple techniques, including scRNA-seq and IHC on paired samples, led to the same conclusion by showing that the ECM-myCAF content is maintained at a high level in patients with chemoresistant high-grade serous ovarian cancer (57). Importantly, spatial transcriptomic analysis of breast cancer samples showed that ECM-myCAFs spatially localize in close proximity to breast cancer cells *in vivo* (28), clearly evidencing a cancer cell–to–ECM-myCAF cross-talk. In agreement with these observations, treatment-resistant epithelial cells from HER2^+^ breast cancer are also found in close proximity to SMA^+^ CAFs (ECM-myCAFs being SMA^+^ myofibroblasts; ref. 68), highlighting a possible link between *in vivo* cancer proximity to these specific CAFs and the acquisition of chemoresistance features. In addition to the study of a cohort of patients with TNBC, we exploited ToC technology to move from clinical correlations to experimental validation of ECM-myCAF–induced chemoprotection and investigation of the underlying molecular mechanism. ToCs contribute to significant advances in cancer research by providing original solutions to the historical limitations of 2D culture and animal models (35–38). Moreover, evaluating the impact of patient-derived stromal components in a timely manner on ToC holds great potential to advance personalized medicine (38, 41, 69–71). We cocultured primary breast ECM-myCAFs with TNBC cells in ToCs under doxorubicin and paclitaxel treatments. ECM-myCAFs induced robust TNBC cell resistance that could be precisely quantified by image analysis of ToC videos. Intriguingly, ECM-myCAFs induced chemoprotection at very early time points of treatment, indicating a priming mechanism by ECM-myCAFs on TNBC cells. Consistently, an advanced deep learning image analysis approach showed that the cell fate of cancer cells—being alive or dead—is determined by the proximity of ECM-myCAFs to cancer cells at early time points of treatment (within 8 hours). Thus, ECM-myCAFs send a survival signal to TNBC cells within the first hours of coculture, a secreted signal we call the “kiss-of-life.”

We investigated the underlying kiss-of-life mechanism by combining scRNA-seq data, functional assays, and gene silencing and by integrating patient and ToC data. We discovered the key role of *G0S2*, a mitochondrial protein (46, 49) that was previously shown to regulate apoptosis (46, 47), cell survival (48), and EMT (48, 50). More specifically, our transcriptomic analysis of chemoresistant patients and TNBC cells upon ECM-myCAF ToC coculture revealed upregulation of several processes in which G0S2 has been previously implied, including mitochondrial oxidative phosphorylation (49). G0S2 was recently shown to exhibit a dual role in breast cancer cells, with an antiproliferative role in estrogen receptor–positive breast cancer by reducing the estrogen signaling pathway and a protumorigenic function in estrogen receptor–negative breast cancer through stimulation of cell proliferation, EMT, and migration (50). To our knowledge, nothing has been reported about the role of G0S2 in anticancer therapy resistance. In ToC assays, G0S2 silencing completely abolished the chemoprotective effects of ECM-myCAFs, indicating that upregulation of G0S2 by ECM-myCAFs is required to decrease drug-induced apoptosis. Consistently, in the absence of the drug, another study showed a prosurvival function for G0S2 in TNBC cells (48). Finally, we provided evidence linking ECM-myCAFs to G0S2 upregulation in TNBC cells via the secretion of soluble factors and activation of SRC kinases. The secretome of activated CAFs (16) contains cytokines that are known to stimulate SRC activity, including TGFβ, IL6, FGF1, FGF2, CXCL12, and HGF. We analyzed the expression at the mRNA level of these ligands in ECM-myCAFs in our scRNA-seq ToC datasets, as well as the expression of their corresponding receptors in TNBC cells. There were only two ligand–receptor pairs expressed at detectable levels: HGF–MET and FGF2–FGFR1. We therefore speculate that HGF and FGF2, and possibly other soluble molecules, mediate the effects observed in our CAF cancer cocultures. Moreover, besides G0S2 and SRC kinase, other intracellular mediators contributing to ECM-myCAF–driven chemoresistance remain to be elucidated.

In conclusion, all these findings together led us to propose a potential mechanistic model for ECM-myCAF–promoted TNBC chemoresistance (Fig. 8). Secreted ECM-myCAF factors (such as IL6 and HGF) increase the activity of SRC family kinases in TNBC cells, upregulating TFs (such as IRF1, STAT3, FOSL2, FOS, CEBPB, JUN, BHLHE40, and RELA) that positively regulate *G0S2* transcription and increase G0S2 protein levels in TNBC cells, leading to resistance to chemotherapy treatment. Importantly, we showed that inhibition of SRC kinases by dasatinib completely abolished the chemoprotective effect of ECM-myCAFs on cancer cells, pointing out a potential clinical strategy to overcome ECM-myCAF–induced cancer chemoresistance. These results contribute to building a better understanding of CAF-dependent chemotherapy resistance in TNBC and may lead to the conception of ECM-myCAF–targeting strategies for future translational applications. More broadly, the ECM-myCAF population is detected not only in high quantities in breast cancers but also in several other cancer types, including ovarian, lung, and head and neck cancers (10, 28, 57). Thus, targeting ECM-myCAFs might help improve patient survival in multiple cancer types.

## Supplementary Material

Video S1 ToC mono-culture of MDA-MB-231, time length 72h, no treatment. Cancer cells are stained in red, cells turning green indicate apoptotic cells. Scale bar = 100 μm.

Video S2 ToC mono-culture of MDA-MB-231, time length 72h, 2μM Doxorubicin. Scale bar = 100 μm.

Video S3 ToC co-culture of MDA-MB-231 with ECM-myCAF, time length 72h, no treatment Scale bar = 100 μm.

Video S4 ToC co-culture of MDA-MB-231 with ECM-myCAF, time length 72h, 2μM Doxorubicin. Scale bar = 100 μm.

Video S5 ToC mono-culture of MDA-MB-231, time length 72h, 1μM Paclitaxel. Scale bar = 100 μm.

Video S6 ToC co-culture of MDA-MB-231 with ECM-myCAF, time length 72h, 1μM Paclitaxel. Scale bar = 100 μm.

Video S7 ToC mono-culture of MDA-MB-436, time length 72h, no treatment. Scale bar = 100 μm.

Video S8 ToC co-culture of MDA-MB-436 and ECM-myCAF, time length 72h, no treatment. Scale bar = 100 μm.

Video S9 ToC mono-culture of MDA-MB-436, time length 72h, 2μM Doxorubicin. Scale bar = 100 μm.

Video S10 ToC co-culture of MDA-MB-436 and ECM-myCAF, time length 72h, 2μM Doxorubicin. Scale bar = 100 μm

Video S11 ToC mono-culture of MDA-MB-436, time length 72h, 1μM Paclitaxel. Scale bar = 100 μm.

Video S12 ToC co-culture of MDA-MB-436 and ECM-myCAFs, time length 72h, 1μM Paclitaxel. Scale bar = 100 μm.

Video S13 ToC mono-culture of primary ECM-myCAFs (from patient #3), time length 72h, no treatment, cells turning green indicate apoptotic cells (Cell event caspase 3/7 reporter), scale bar = 100 μm

Video S14 ToC mono-culture of primary ECM-myCAFs (resistant, from patient #3), time length 72h, 2μM Doxorubicin. Scale bar = 100 μm.

Video S15 ToC mono-culture of primary ECM-myCAFs (sensitive, from patient #2), time length 72h, 2μM Doxorubicin. Scale bar = 100 μm.

Video S16 ToC mono-culture of primary ECM-myCAFs (from patient #10) time length 72h, 1μM Paclitaxel. Scale bar = 100 μm.

Video S17 ToC co-culture of MDA-MB-231 cells and ECM-myCAFs, time length 72h, 2μM Doxorubicin. Cells were segmented using a Convolutional Neural Network (CNN). Red = cancer cells. Blue = ECM-myCAF. Scale bar = 100 μm.

Figure S1 Cell types and cell states identified in TNBC patients before and after chemotherapy, stratified by chemo-sensitive and chemo-resistant cases

Figure S2 Identity of ECM-myCAFs before and after ToC culture and their chemoprotective effect on MDA-MB-436 cells

Figure S3 Metascape analyses of cancer cells from TNBC patients and of cancer cells and ECM-myCAFs derived from the ToC system

Figure S4 G0S2 expression is elevated in cancer cells following direct ECM-myCAF co-culture in 2D conditions

Figure S5 Kinase activity profiles in MDA-MB-231 and MDA-MB-436 cells upon ECM-myCAF transwell co-culture

Figure S6 Kinetics of SRC activation and G0S2 expression in MDA-MB-231 cells upon ECM-myCAF co-culture and/or Doxorubicin treatment, as well as the effects of G0S2 knockdown on SRC activation in MDA-MB-436 cells

Table S1 Detailed information on the SCANDARE cohort

## Data Availability

The data generated in this study are publicly available in the European Genome-phenome Archive at EGAS50000000886 (scRNA-seq from ToC) and EGAS50000000970 (bulk RNA-seq from the TNBC SCANDARE Curie cohort). Controlled access is required as raw data contain potentially identifiable patient information and can be granted through the European Genome-phenome Archive following completion of an institutional data transfer agreement. Processed RNA-seq data from the TNBC SCANDARE Curie cohort, together with clinical data [time points of the sampling (pre-/post-treatment), chemoresistance, or chemosensitivity of the patients], are available in the Figshare data repository at https://doi.org/10.6084/m9.figshare.28485827. Codes developed for this study are deposited in the Zenodo open-access repository (RRID: SCR_004129) at https://doi.org/10.5281/zenodo.14844774. All other raw data generated in this study are available from the corresponding author upon request.
