# Supplementary material for: An Extracellular Matrix–Producing Subset of Cancer-Associated Fibroblasts Drives Chemoresistance in Breast Cancer via SRC Activation and G0S2 Upregulation
Source: Cancer Res. 2025 Nov 12;86(4):1054–72. doi: 10.1158/0008-5472.CAN-25-0966 (PMC13053057; doi:10.1158/0008-5472.CAN-25-0966)
Supplement: Figure S1 — Cell types and cell states identified in TNBC patients before and after chemotherapy, stratified by chemo-sensitive and chemo-resistant cases [file can-25-0966_figure_s1_suppsf1.pdf]

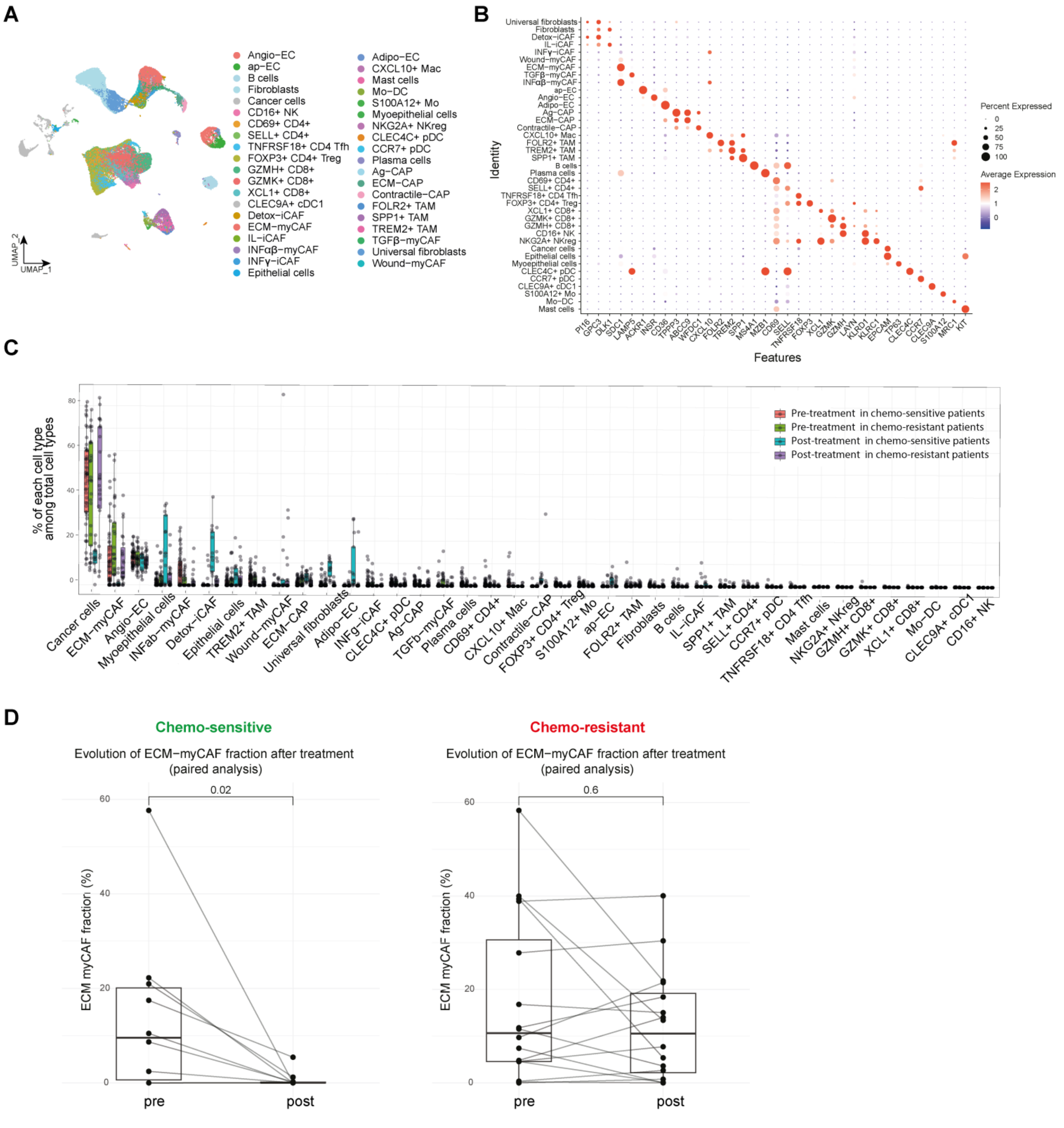

Supplementary Figure S1. (A) Uniform manifold approximation and projection (UMAP) of 63,374 single cells from 43 BC patients and 9 healthy donors (reduction mammoplasties), which compose a comprehensive BC cellular atlas and identify 39 different cell types and cell states (derived from Croizer et al., Nature Comm, 2024). **(B)** Dot plot showing expression of specific genes in each of the 39 cell types and states from the BC atlas. Colors show the mean expression level across all cells. Sizes of dots represent the percentages of cells within a cell type expressing one given gene. **(C)** Cell type fraction (%) across chemo-sensitive and chemo-resistant patients, pre-/post chemotherapy treatment (N = 114). **(D)** Paired evolution of ECM-myCAF fraction after treatment in chemo-sensitive **(LEFT)** and chemo-resistant **(RIGHT)** patients at individual patient level.
