# Supplementary material for: An Extracellular Matrix–Producing Subset of Cancer-Associated Fibroblasts Drives Chemoresistance in Breast Cancer via SRC Activation and G0S2 Upregulation
Source: Cancer Res. 2025 Nov 12;86(4):1054–72. doi: 10.1158/0008-5472.CAN-25-0966 (PMC13053057; doi:10.1158/0008-5472.CAN-25-0966)
Supplement: Figure S2 — Identity of ECM-myCAFs before and after ToC culture and their chemoprotective effect on MDA-MB-436 cells [file can-25-0966_figure_s2_suppsf2.pdf]

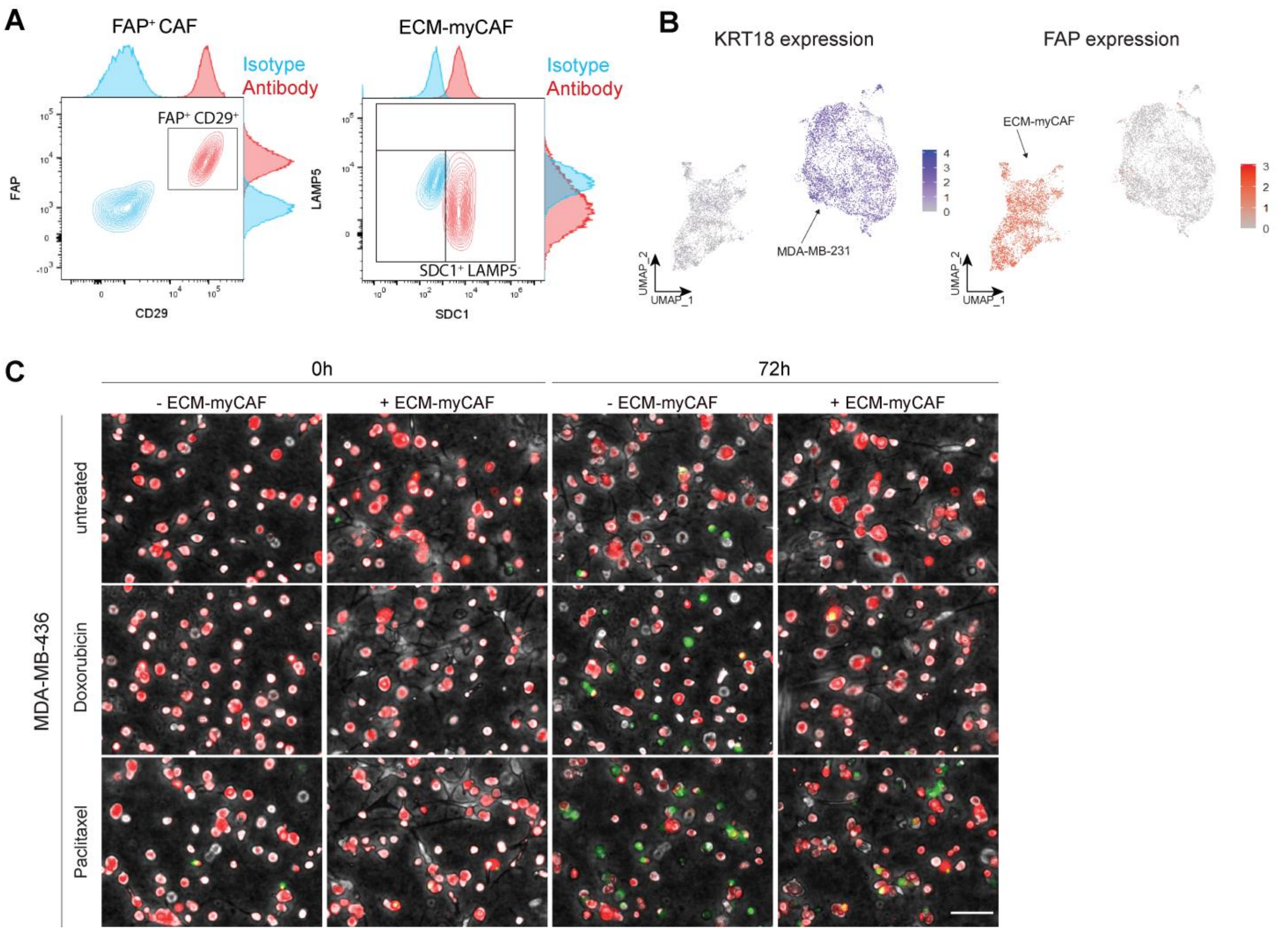

Supplementary Figure S2. (A) Identity of ECM-myCAF after explant culture assessed by flow cytometry. **(B)** UMAP from scRNA-seq data (n=9,651 total cells; 3,866 ECM-myCAF and 5,785 MDA-MB-231) showing KRT18 (**LEFT**) and FAP (**RIGHT**) expression, highlighting the cancer cells and ECM-myCAF populations, from two different patient-derived ToC after mono- and co-culture. **(C)** Representative images of MDA-MB-436 ± ECM-myCAF under Doxorubicin or Paclitaxel treatment at acquisition start (0h) and 72h post culture in ToC. Scale bar = 100µm.
