# Supplementary material for: An Extracellular Matrix–Producing Subset of Cancer-Associated Fibroblasts Drives Chemoresistance in Breast Cancer via SRC Activation and G0S2 Upregulation
Source: Cancer Res. 2025 Nov 12;86(4):1054–72. doi: 10.1158/0008-5472.CAN-25-0966 (PMC13053057; doi:10.1158/0008-5472.CAN-25-0966)
Supplement: Figure S3 — Metascape analyses of cancer cells from TNBC patients and of cancer cells and ECM-myCAFs derived from the ToC system [file can-25-0966_figure_s3_suppsf3.pdf]

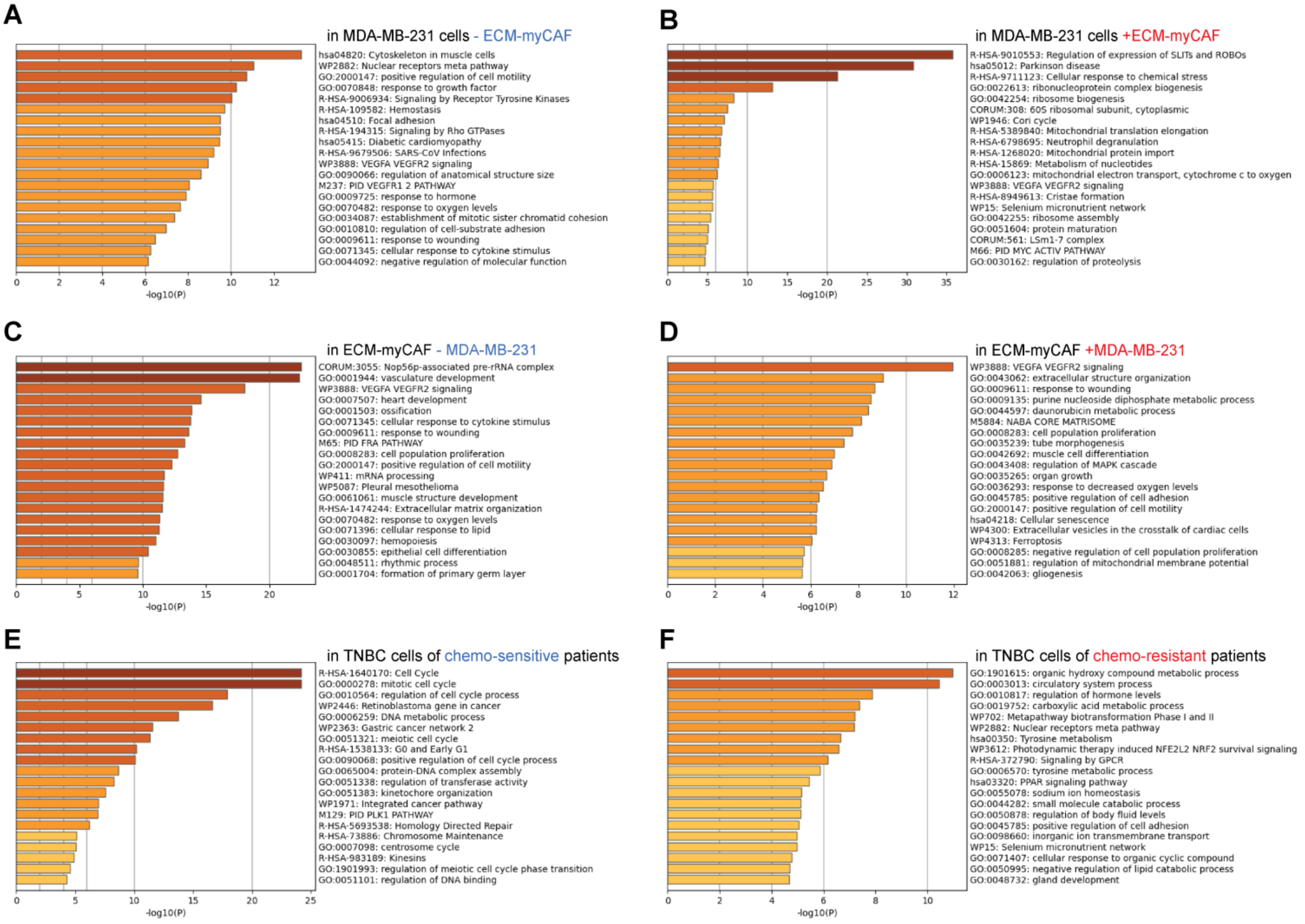

Supplementary Figure S3. Analysis of transcriptomic data from ToC-derived MDA-MB-231 cancer cells and ECM-myCAF and from chemo-sensitive and chemo-resistant TNBC patients. **(A, B)** Metascape analysis based on upregulated genes in MDA-MB-231 cells after ToC mono-culture (A) or co-culture with ECM-myCAF (B). **(C, D)** Metascape analysis based on upregulated genes in ECM-myCAF after ToC mono-culture **(**C) or co-culture with MDA-MB-231 cells (D). **(E, F)** Metascape analysis based on upregulated genes in TNBC cells from chemo-sensitive patients (E) and chemo-resistant patients (F) at baseline.
