# Supplementary material for: An Extracellular Matrix–Producing Subset of Cancer-Associated Fibroblasts Drives Chemoresistance in Breast Cancer via SRC Activation and G0S2 Upregulation
Source: Cancer Res. 2025 Nov 12;86(4):1054–72. doi: 10.1158/0008-5472.CAN-25-0966 (PMC13053057; doi:10.1158/0008-5472.CAN-25-0966)
Supplement: Figure S4 — G0S2 expression is elevated in cancer cells following direct ECM-myCAF co-culture in 2D conditions [file can-25-0966_figure_s4_suppsf4.pdf]

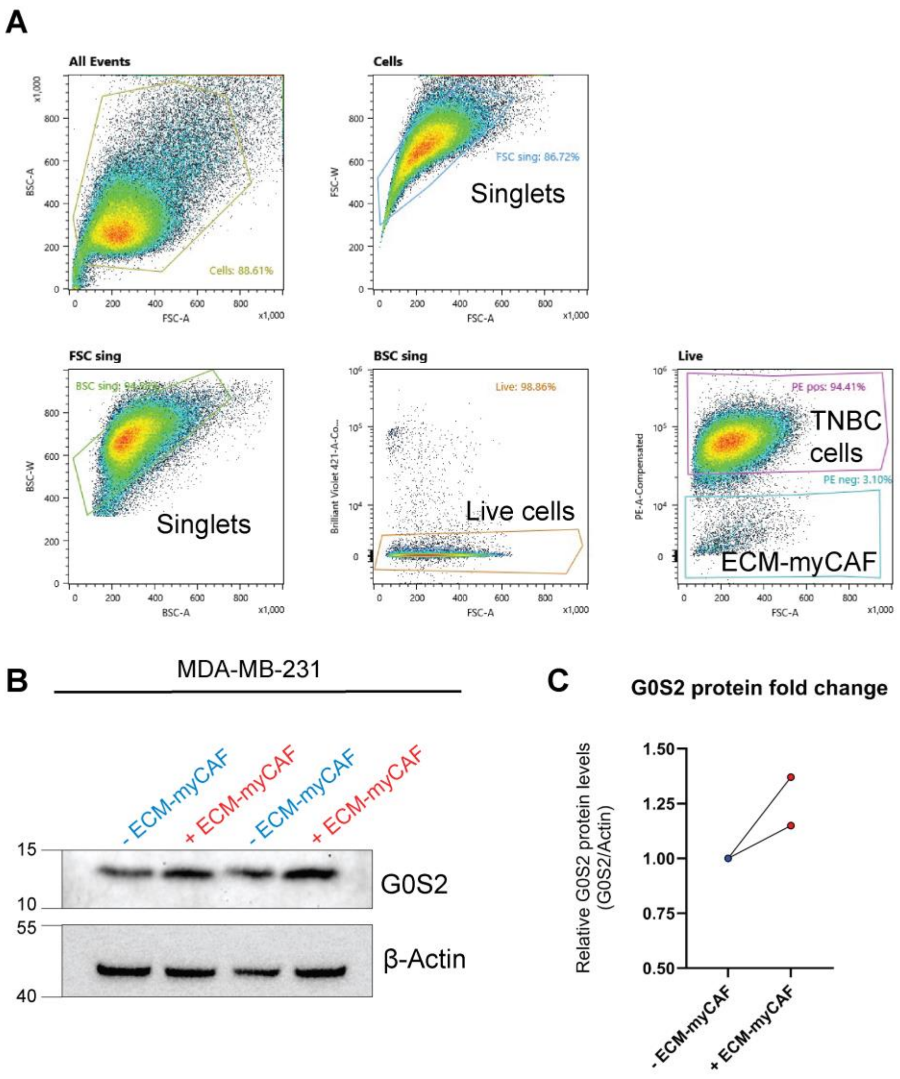

Supplementary Figure S4: Analysis of G0S2 protein levels in MDA-MB-231 cells upon co-culture with ECM-myCAFs in standard 2D dishes. MDA-MB-231 cells were stained with 5µM CellTrace™ Yellow (Invitrogen, #C34567) according to manufacturer's protocol. Then, $2x10^6$ TNBC cells were seeded in tissue culture plates (10-cm) with $8x10^5$ ECM-myCAFs (3.5:1 ratio). After 72h of (co-)culture, cells were collected in PBS containing 10% FBS and 2mM EDTA prior to FACS analysis. Only live cells were collected. CellTrace™ yellow stained TNBC cells were separated from unstained ECM-myCAF. After sorting, cells were centrifuged and immediately used for protein isolation. **(A)** Representative FACS gating strategy to separate TNBC cells from ECM-myCAF after direct co-culture. **(B)** Western blot of G0S2 levels in MDA-MB-231 cells after mono- or direct co-culture with 2 different patient derived ECM-myCAFs. **(C)** Quantification of (B). G0S2 levels were normalized to G0S2 levels in MDA-MB-231 in mono-culture.
