# Supplementary material for: An Extracellular Matrix–Producing Subset of Cancer-Associated Fibroblasts Drives Chemoresistance in Breast Cancer via SRC Activation and G0S2 Upregulation
Source: Cancer Res. 2025 Nov 12;86(4):1054–72. doi: 10.1158/0008-5472.CAN-25-0966 (PMC13053057; doi:10.1158/0008-5472.CAN-25-0966)
Supplement: Figure S5 — Kinase activity profiles in MDA-MB-231 and MDA-MB-436 cells upon ECM-myCAF transwell co-culture [file can-25-0966_figure_s5_suppsf5.pdf]

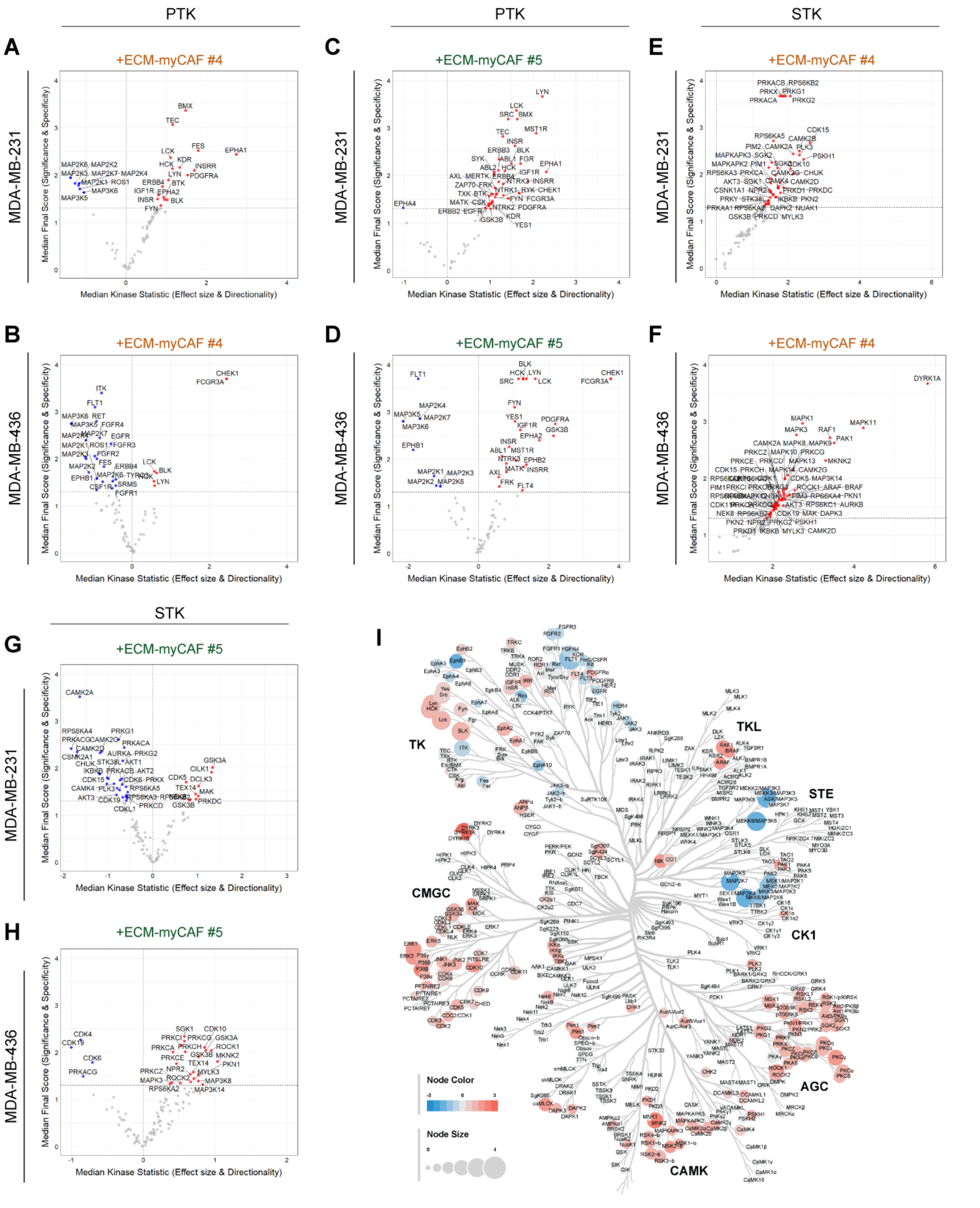

Supplementary Figure S5. **(A-H)** Volcano plot depicting differentially regulated PTK and STK kinase activity in MDA-MB-231 and MDA-MB-436 cells upon co-culture with ECM-myCAF derived from patient #4 (A, B, E, F) and patient #5 (C, D, G, H). **(I)** Kinome tree depicting up- and downregulation of different kinase family activities in MDA-MB-436 cells upon ECM-myCAF co-culture (combining patient #4 and #5).
