# Supplementary material for: An Extracellular Matrix–Producing Subset of Cancer-Associated Fibroblasts Drives Chemoresistance in Breast Cancer via SRC Activation and G0S2 Upregulation
Source: Cancer Res. 2025 Nov 12;86(4):1054–72. doi: 10.1158/0008-5472.CAN-25-0966 (PMC13053057; doi:10.1158/0008-5472.CAN-25-0966)
Supplement: Figure S6 — Kinetics of SRC activation and G0S2 expression in MDA-MB-231 cells upon ECM-myCAF co-culture and/or Doxorubicin treatment, as well as the effects of G0S2 knockdown on SRC activation in MDA-MB-436 cells [file can-25-0966_figure_s6_suppsf6.pdf]

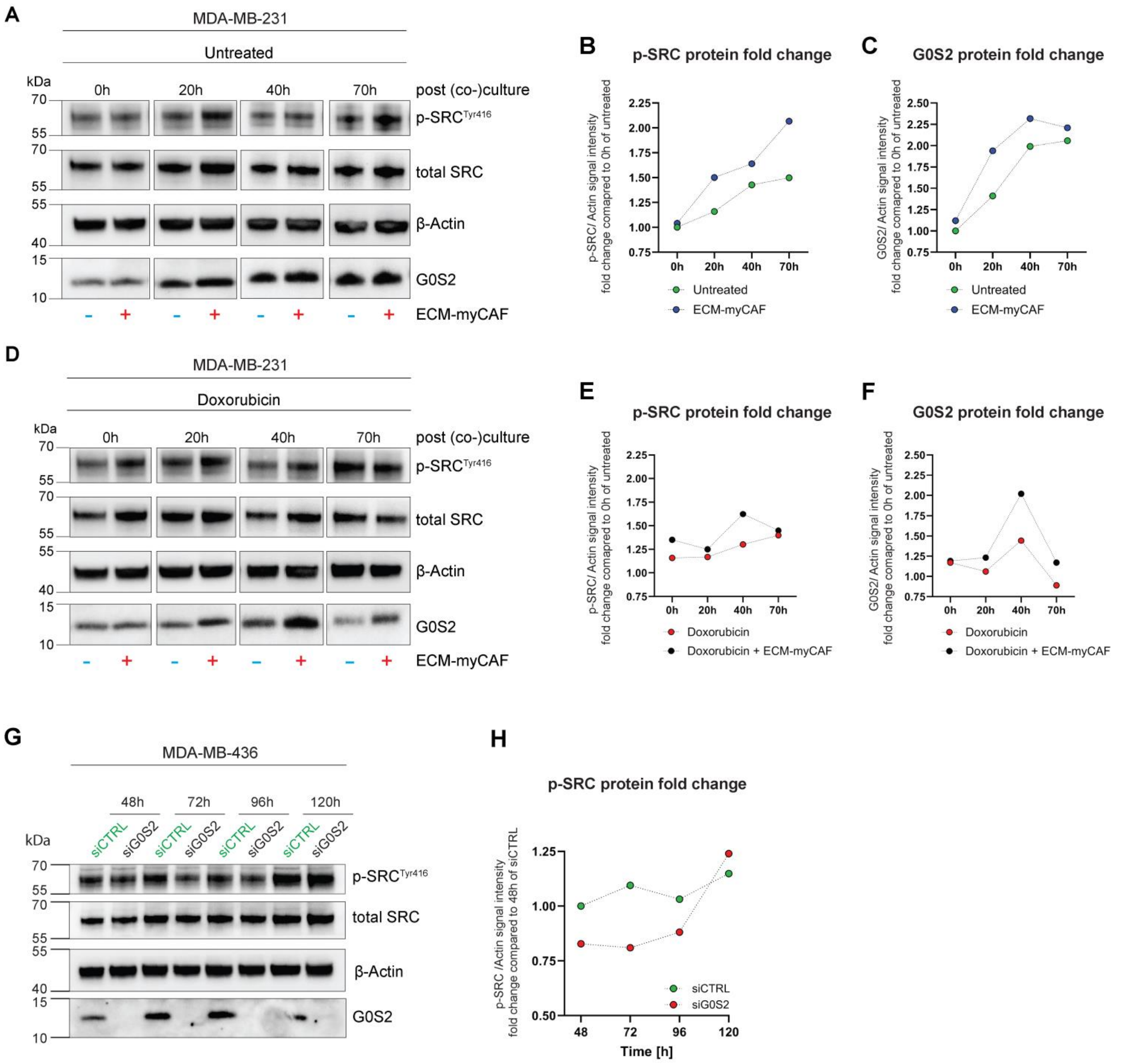

Supplementary Figure S6. (A) Western blot showing p-SRC, total SRC, G0S2 and β-Actin levels in MDA-MB-231 cells ± ECM-myCAF after 0h, 20h, 40h or 70h post (co-)culture. **(B, C)** Quantification of (A). p-SRC and G0S2 levels were normalized to p-SRC and G0S2 levels of untreated TNBC cells at t0h. **(D)** Western blot showing p-SRC, total SRC, G0S2 and β-Actin levels in MDA-MB-231 cells in the presence of 100nM Doxorubicin ± ECM-myCAF after 0h, 20h, 40h or 70h post (co-)culture and treatment initiation. **(E, F)** Quantification of (D). p-SRC and G0S2 levels were normalized to p-SRC and G0S2 levels of untreated TNBC cells at t0h **(G, H)** Western blot showing p-SRC, total SRC, G0S2 and β-Actin protein levels in (G) MDA-MB-436 cells 48h, 72h, 96h and 120h silenced (siG0S2) or not (siCTRL) for G0S2. (H) Quantification of (G). p-SRC levels of siCTRL and siG0S2 cells were normalized to p-SRC levels of siCTRL cells at 48h post culture.
