# Supplementary material for: An Extracellular Matrix–Producing Subset of Cancer-Associated Fibroblasts Drives Chemoresistance in Breast Cancer via SRC Activation and G0S2 Upregulation
Source: Cancer Res. 2025 Nov 12;86(4):1054–72. doi: 10.1158/0008-5472.CAN-25-0966 (PMC13053057; doi:10.1158/0008-5472.CAN-25-0966)
Supplement: Table S1 — Detailed information on the SCANDARE cohort [file can-25-0966_table_s1_suppst1.pdf]

		All patients	chemo-sensitive patients	chemo-resistant patients
**Number of patients**		88	52 (59%)	36 (41%)
**Age at Inclusion**	min - max (median)	24 - 74 (45)	24 - 68 (47)	25 - 74 (44)
**Classification of cancer**				
**Tumor (T)**	T1	8 (9.1%)	6 (11.5%)	2 (5.6%)
	T2	58 (65.9%)	32 (61.5%)	26 (72.2%)
	T3	15 (17%)	9 (17.3%)	6 (16.7%)
	T4	6 (6.8%)	4 (7.7%)	2 (5.6%)
	NA	1 (1.1%)	1 (1.9%)	0 (0%)
**Lymph node (N)**	N0	48 (54.5%)	31 (59.6%)	17 (47.2%)
	N1	32 (36.4%)	14 (26.9%)	18 (50%)
	N3	7 (8%)	6 (11.5%)	1 (2.8%)
	NA	1 (1.1%)	1 (1.9%)	0 (0%)
**Metastasis (M)**	M0	85 (96.6%)	50 (96.2%)	35 (97.2%)
	M1	1 (1.1%)	1 (1.9%)	0 (0%)
	MX	1 (1.1%)	0 (0%)	1 (2.8%)
	NA	1 (1.1%)	1 (1.9%)	0 (0%)
**Histological type**	Ductal	82 (93.2%)	47 (90.4%)	35 (97.2%)
	Other	5 (5.7%)	4 (7.7%)	1 (2.8%)
	NA	1 (1.1%)	1 (1.9%)	0 (0%)
**Elston-Ellis (EE) grade**	2	27 (30.7%)	11 (21.2%)	16 (44.4%)
	3	55 (62.5%)	38 (73.1%)	17 (47.2%)
	NA	6 (6.8%)	3 (5.8%)	3 (8.3%)
**Neoadjuvant chemotherapy**	Anthracyclines + Taxanes	80 (90.9%)	49 (94.2%)	31 (86.1%)
	Others	6 (6.8%)	2 (3.8%)	4 (11.1%)
	NA	2 (2.3%)	1 (1.9%)	1 (2.8%)
**Type of surgery**	Lumpectomy	55 (62.5%)	38 (73.1%)	17 (47.2%)
	Mastectomy	31 (35.2%)	13 (25%)	18 (50%)
	NA	2 (2.3%)	1 (1.9%)	1 (2.8%)
**Overall survival**	Mean (months)	55.8	60.11	49.68
	number of event	18 (20.5%)	5 (9.6%)	13 (36.1%)
**Disease-free survival**	Mean (months)	51.6	57	44
	number of event	21 (23.9%)	6 (11.5%)	15 (41.7%)
**Residual Cancer Burden (RCB)**	0	38 (43.2%)	38 (73.1%)	0 (0%)
	I	13 (14.8%)	13 (25%)	0 (0%)
	II	24 (27.3%)	0 (0%)	24 (66.7%)
	III	12 (13.6%)	0 (0%)	12 (33.3%)

Supplementary Table S1: Description of the SCANDARE Curie cohort of patients with TNBC.
